# Supplementary material for: Effects of voluntary exercise, diet, and selenium on hypothalamic adult neurogenesis
Source: Stem Cell Reports. 2026 Jun 11;21(7):102953. doi: 10.1016/j.stemcr.2026.102953 (PMC13385432; doi:10.1016/j.stemcr.2026.102953)
Supplement: Document S1. Figures S1–S7 [file mmc1.pdf]

Stem Cell Reports, Volume *21*

Supplemental Information

Effects of voluntary exercise, diet, and selenium on hypothalamic adult neurogenesis

Sara K.M. Jörgensen, Rachel E. Martin, Andrew Want, James Morgan, and David Petrik

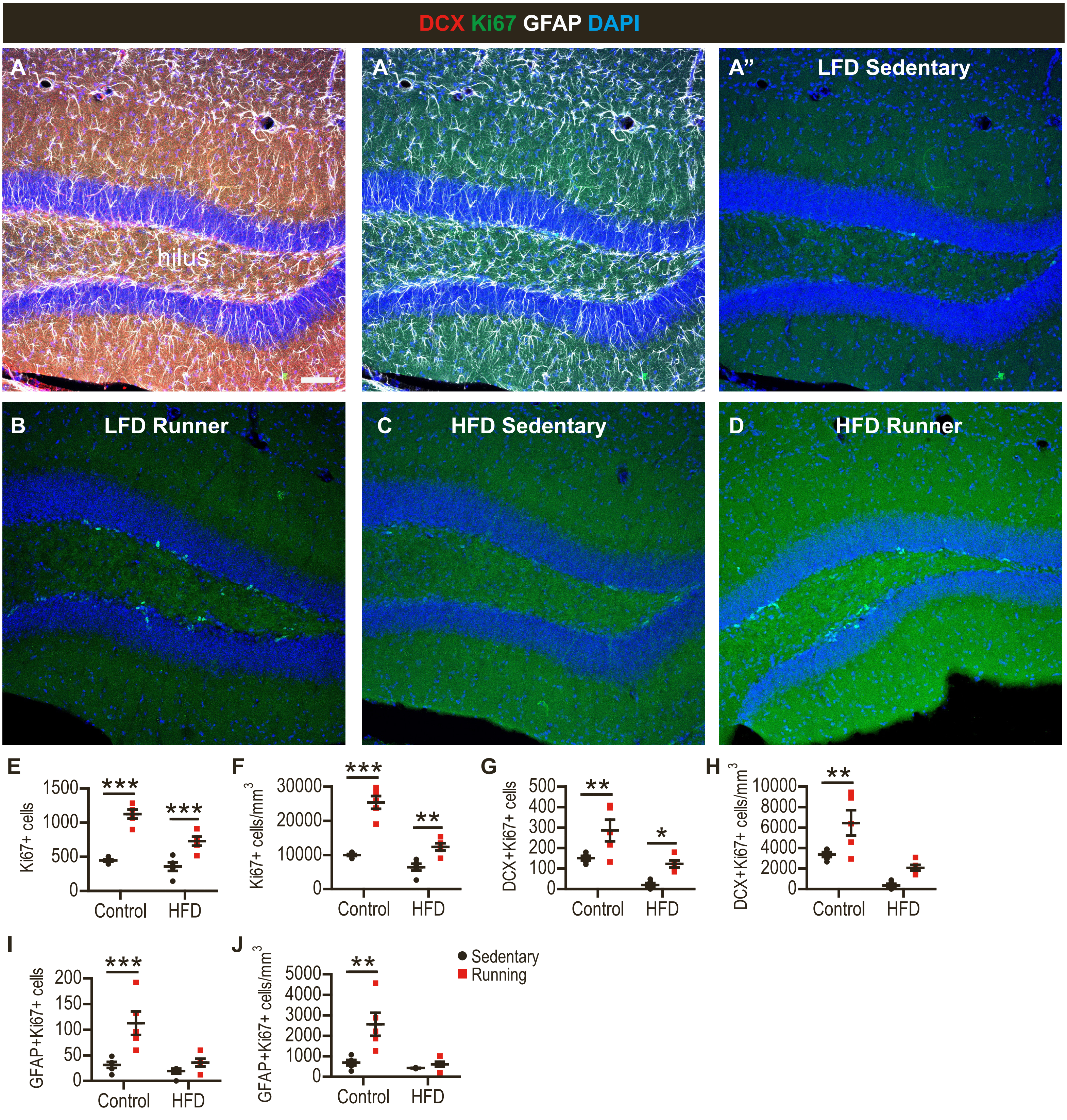

Figure S1. Running increases cell proliferation in the SGZ in both Control and HFD, related to Figure 2

(A-D) Representative confocal images of SGZ stained for DCX, Ki67 and GFAP in Control Sedentary (A), Control Runner (B), HFD Sedentary (C), and HFD Runner (D).

(E-J) Quantification of absolute number and density of proliferating cells (E-F), proliferating neuroblasts (G-H), and proliferating neural stem cells (I-J) in the SGZ.

N = 5/group. Scale bar: 50 μm. Data information: Two-Way ANOVA (panel E: exercise $F(1,16) = 85.87$, $p < 0.0001$, diet $F(1,16) = 18.27$, $p = 0.0006$, interaction $F(1,16) = 7.33$, $p < 0.016$; F: exercise $F(1,16) = 78.35$, $p < 0.0001$, diet $F(1,16) = 47.40$, $p < 0.0001$, interaction $F(1,16) = 15.38$, $p = 0.0012$; G: exercise $F(1,16) = 17.19$, $p = 0.0008$, diet $F(1,16) = 26.54$, $p < 0.0001$, interaction $F(1,16) = 0.3$, $p = 0.59$; H: exercise $F(1,16) = 13.4$, $p = 0.0021$, diet $F(1,16) = 31.81$, $p < 0.0001$, interaction $F(1,16) = 1.05$, $p = 0.32$; I: exercise $F(1,16) = 15.01$, $p = 0.0013$, diet $F(1,16) = 12.22$, $p = 0.0.003$, interaction $F(1,16) = 6.51$, $p = 0.02$; J: exercise $F(1,15) = 22.78$, $p = 0.0056$, diet $F(1,15) = 26.74$, $p =0.0032$, interaction $F(1,15) = 15.62$, $p = 0.017$).
**$p < 0.01$, ***$p < 0.001$ (Bonferroni's test). Data are presented as mean ± SEM.

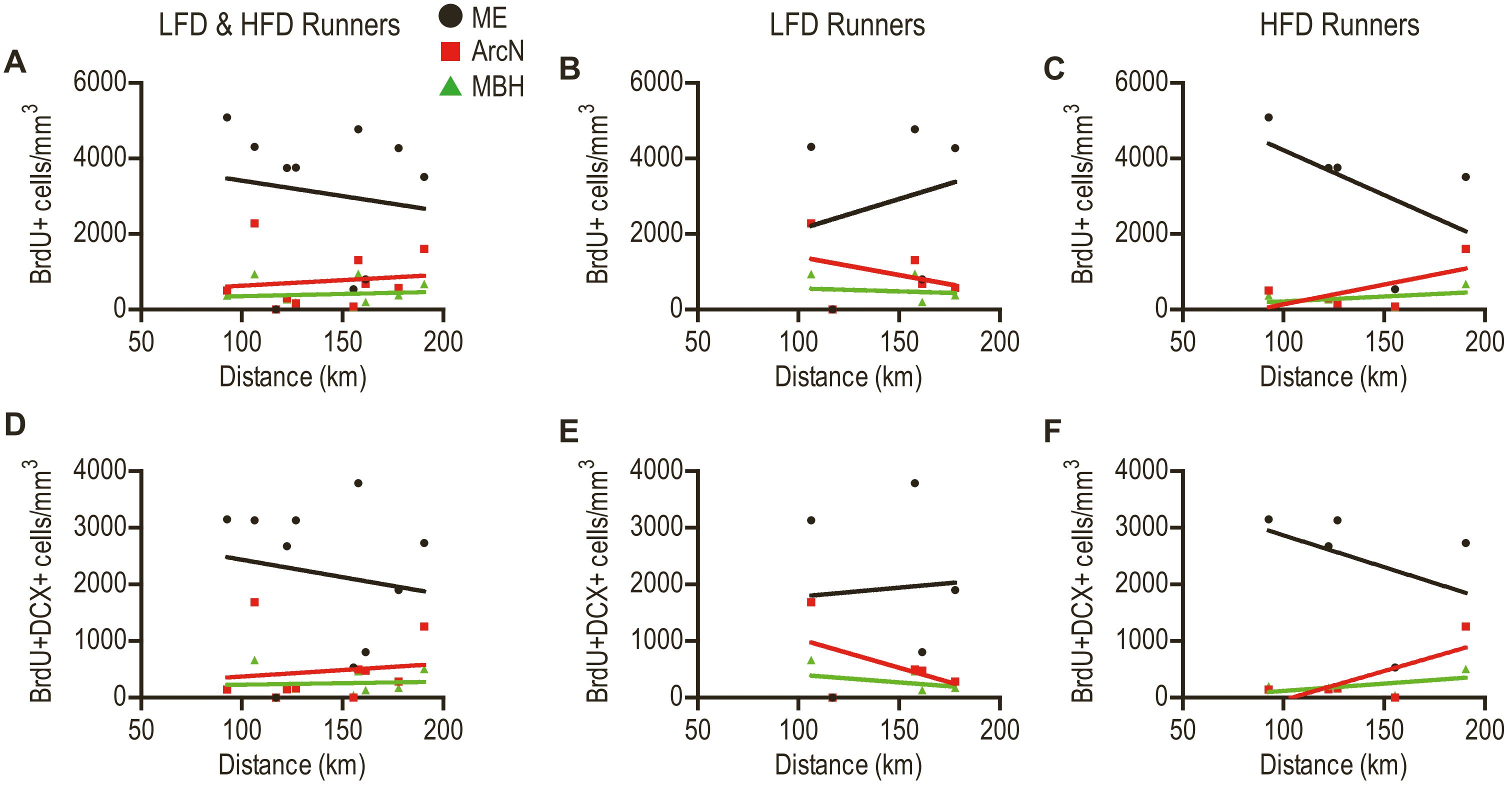

Figure S2. No correlation between running distance and density of cells in different MBH compartments, related to Figure 2 and 3

(A-C) Density of BrdU+ cells as a function of distance ran (kilometres in 14 days) in Control (LFD) and HFD animals combined (A), in Control Runners (B) and in HFD Runners (C).

(D-F) Density of BrdU+DCX+ cells as a function of distance ran (kilometres in 14 days) in Control (LFD) and HFD animals combined (D), in Control Runners (E) and in HFD Runners (F).

Individual points depict the cell density, and the corresponding running distance for each individual animal.

In all graphs, data are depicted for the cell densities in the ME (black circles), the ARH (red squares) and the MBH (green triangles).

Data information: Linear regression (all n.s.).

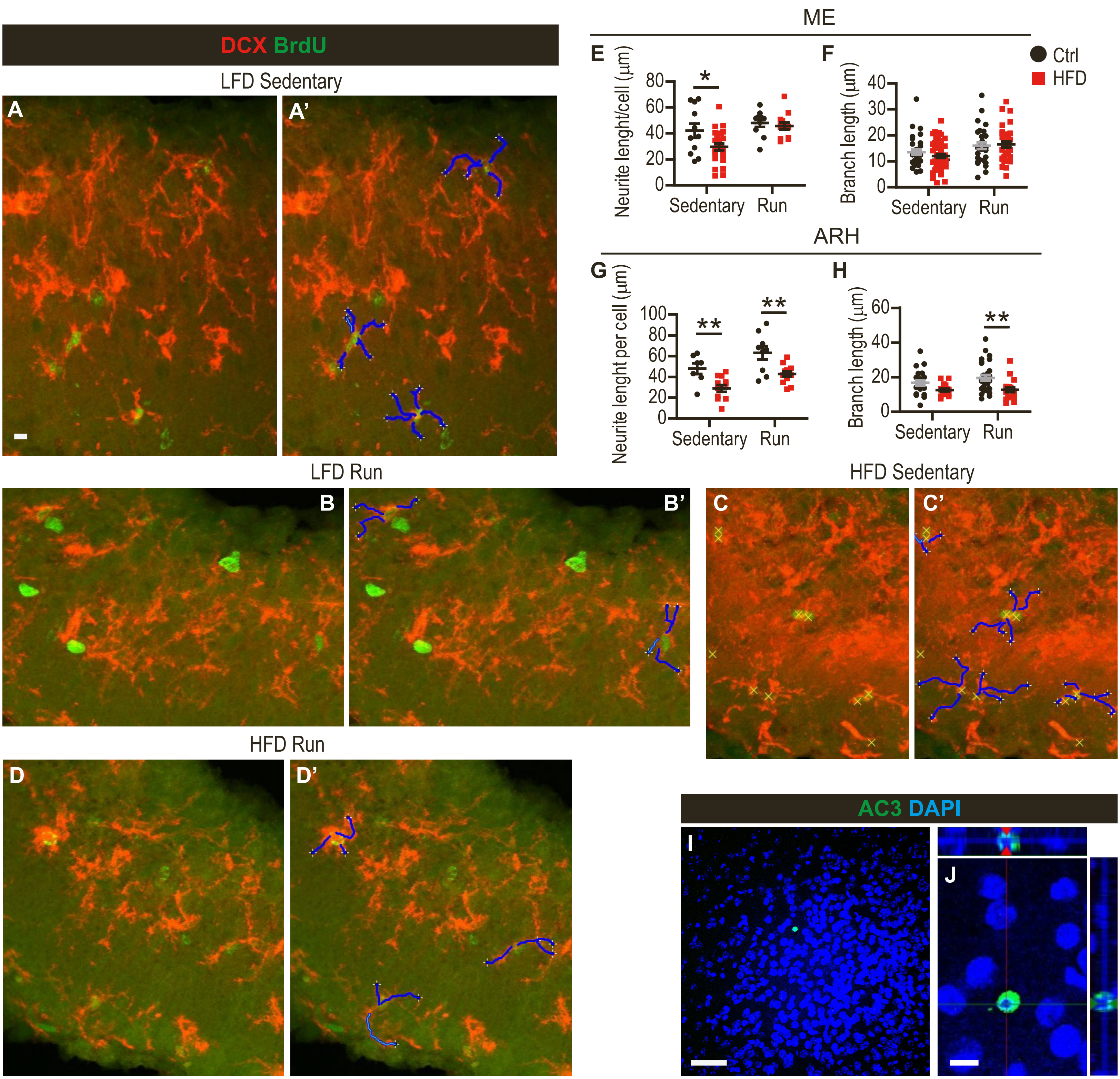

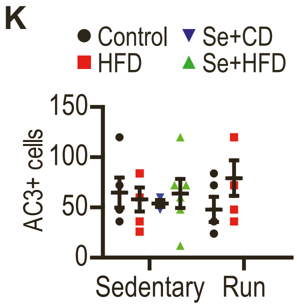

Figure S3. Neurite tracing of BrdU+DCX+ cells and analysis of AC3+ cells at the 2-week time point, related to Figure 2 and 3

(A-D) Examples of BrdU+DCX+ cells in the ME in 4 different treatment groups. Dashed images (e.g., A') show traced neurites.

(E-H) Neurite length per cell (E,G) and branch length (F,H) in the ME (E,F) and the ARH (G,H).

(I) A confocal image of the MBH parenchyma with a AC3+ cell.

(J) An orthogonal projection view of an AC3+ cell (from I).

(J) Quantification of AC3+ cells in different treatment groups (Se+CD = Selenium + Control diet; Se+HFD = Selenium + HFD).

N = 5/group. S.b. = 10 μm (A,J), 50 μm (I).

Data information: Two-Way ANOVA (panel E: exercise $F(1,54) = 9.28$, $p = 0.0036$, diet $F(1:54) = 4.14$, $p = 0.047$, interaction: n.s.; F: exercise $F(1,148) = 11.78$, $p = 0.0008$, diet & interaction: n.s.; G: exercise $F(1,36) = 11.30$, $p = 0.0019$, diet $F(1,36) = 21.03$, $p < 0.0001$, int.: n.s.; H: diet $F(1,85) = 14.41$, $p = 0.0003$, exercise & interaction: n.s), Three-Way ANOVA (panel K: all n.s.).

*$p < 0.05$, *$p < 0.01$ (Bonferroni's test). Data are presented as mean ± SEM.

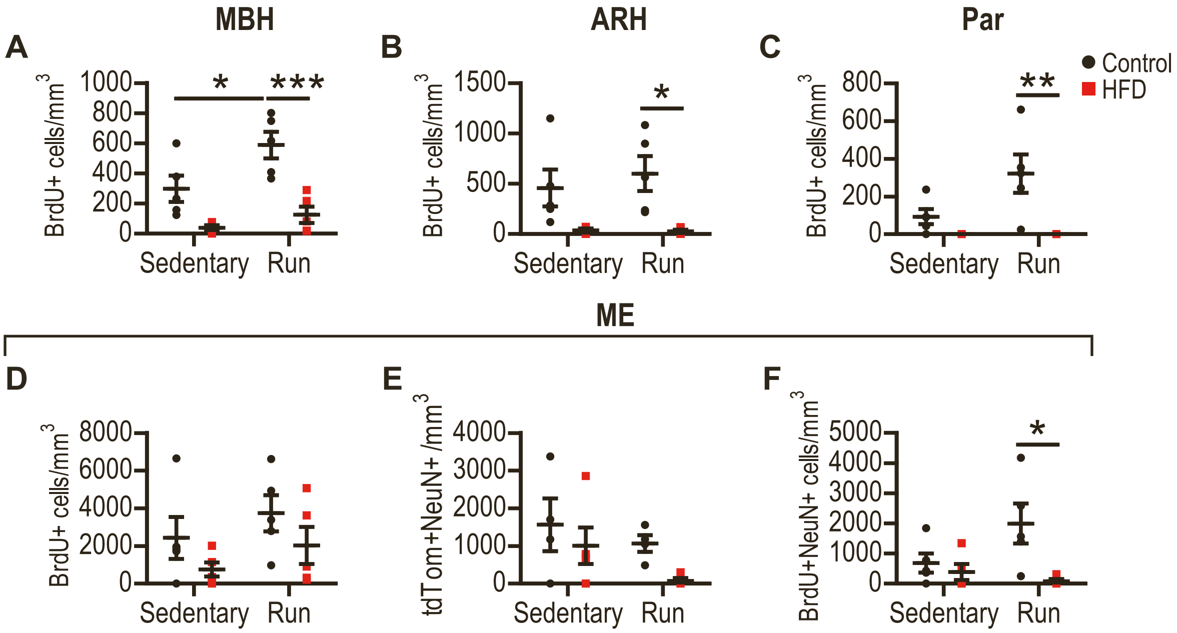

Figure S4. Long-term HFD reduces the density of new cells, related to Figure 4
(A-C) Density of BrdU+ in the MBH (A), the ARH (B), and parenchyma (C).
(D-F) Density of BrdU+ (D), tdTom+NeuN+ (E), and BrdU+NeuN+ (F) cells in the ME.
N = 5/group. Data information: Two-Way ANOVA (panel A: exercise $F(1,15) = 6.75$, $p = 0.02$, diet $F(1,15) = 24.93$, $p = 0.0002$, interaction: n.s.; B: exercise and interaction: n.s., diet $F(1,15) = 13.65$, $p = 0.0022$; C: exercise and interaction: n.s., diet $F(1,15) = 11.03$, $p = 0.0051$; D: all n.s.; E: all n.s.; F: exercise and interaction: n.s., diet $F(1,15) = 7.11$, $p = 0.018$).
*$p < 0.05$, ***$p < 0.001$ (Bonferroni's test). Data are presented as mean ± SEM.

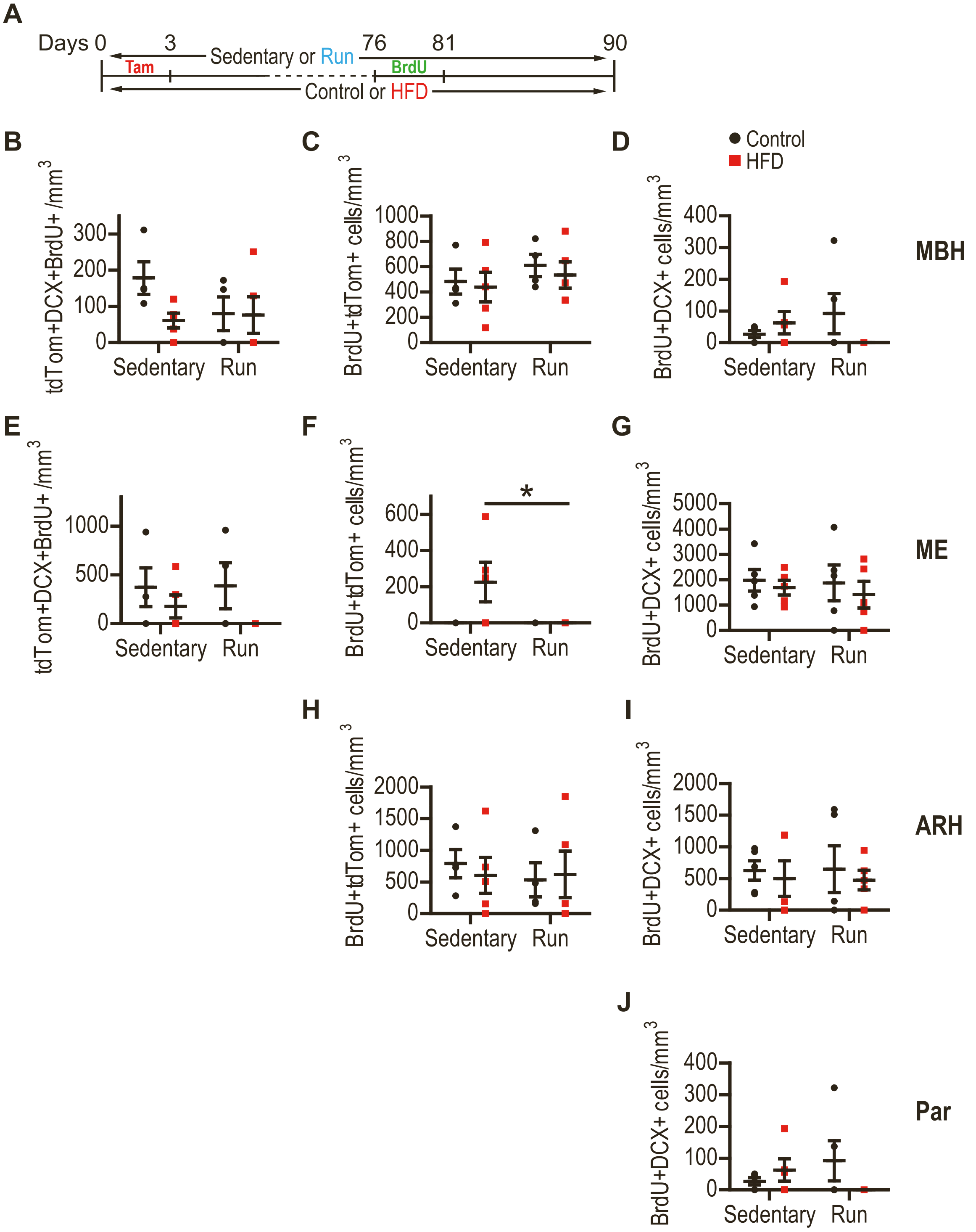

Two-Way ANOVA results

tdTom+DCX+BrdU+				tdTom+BrdU+				DCX+BrdU+				
MBH	ARH	ME	Par	MBH	ARH	ME	Par	MBH	ARH	ME	Par	
n.s.	***	n.s.	***	n.s.	n.s.	n.s.	*	n.s.	n.s.	n.s.	n.s.	Exercise
n.s.	*	n.s.	***	n.s.	n.s.	n.s.	n.s.	n.s.	n.s.	n.s.	n.s.	Diet
n.s.	**	n.s.	***	n.s.	n.s.	n.s.	n.s.	n.s.	n.s.	n.s.	n.s.	Interaction

Figure S5. Long-term running or HFD do not alter the density of BrdU+DCX+ cells, related to Figure 5
(A) A schematic of the protocol.
(B, E) Density of tdTom+DCX+BrdU+ cells in the MBH (B) and the ME (E).
(C, F, H) Density of BrdU+tdTom+ cells in the MBH (C), the ME (F) and the ARH (H).
(D, G, I, J) Density of BrdU+DCX+ cells in the MBH (D), the ME (G), the ARH (I) and the parenchyma (J).
The table depicts statistical significance in all 4 hypothalamic compartments. Two-Way ANOVA: *p < 0.05, **p < 0.01, ***p < 0.001.
Data are presented as mean ± SEM.

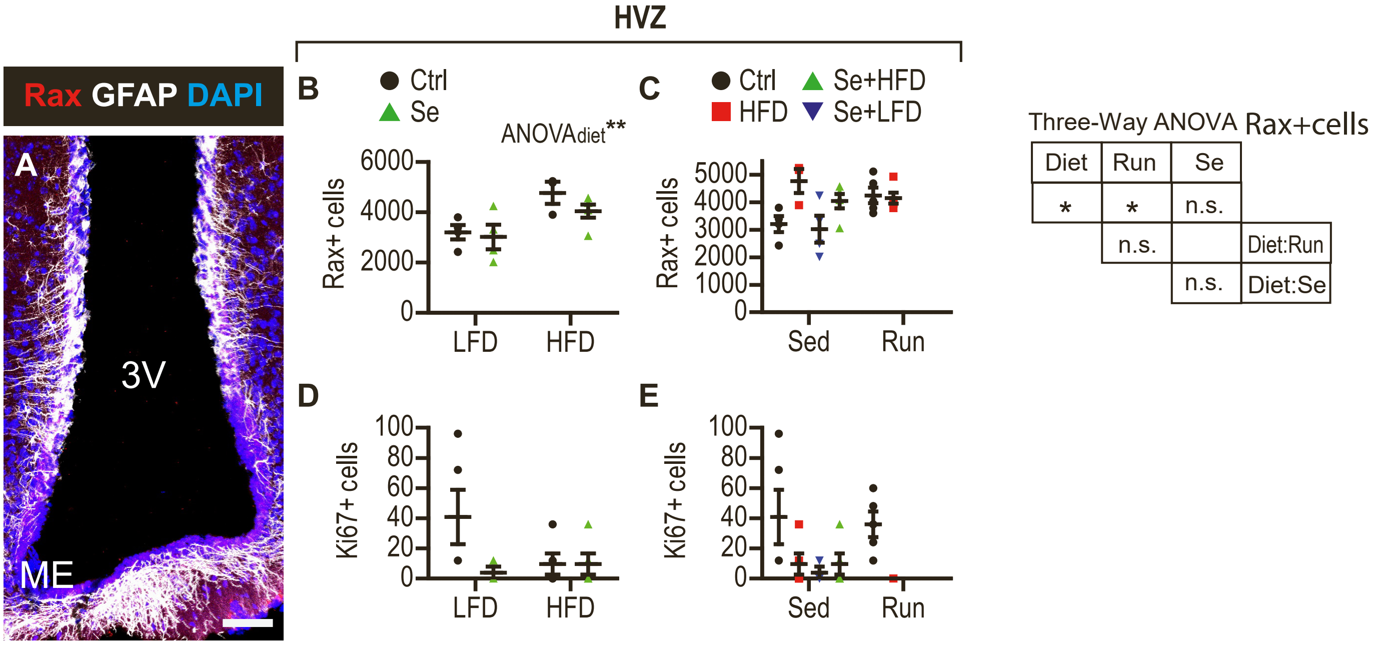

Figure S6. Selenium does not affect the number of HVZ cells, related to Figure 6
(A) A representative confocal image of the HVZ stained as indicated.
(B-C) Quantification of RAX+ cells in the HVZ.
(D-E) Quantification of Ki67+ cells in the HVZ.
N = 5/group. S.b. = 50 μm. Data information: Two-Way ANOVA (panel B: exercise and interaction: n.s., diet $F(1,12) = 12.16$, $p = 0.0045$), Three-Way ANOVA (panel E: selenium $F(1.22) = 7.06$, $p = 0.014$). Three-Way ANOVA table for panel C. (B, D: Bonferroni's test, C, E: Tukey test). Data are presented as mean ± SEM.

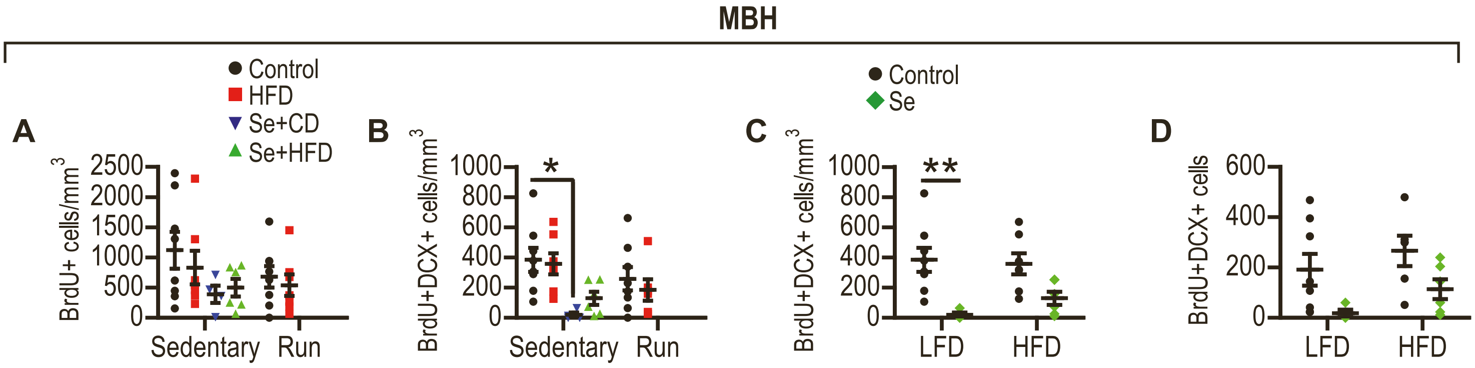

Figure S7. Selenium reduces number of new immature neurons in the MBH, related to Figure 7

(A-D) Quantifications of the density of BrdU+ (A) and BrdU+DCX+ cells (B-C) and the number of BrdU+DCX+ cells (D) in the MBH. N = 4-6/group. Data information: Three-Way ANOVA (panel A: selenium $F(1,34) = 4.28$, $p = 0.046$, all other: n.s.; B: selenium $F(1,33) = 14.26$, $p = 0.00063$, all other: n.s.), Two-Way ANOVA (panel C: selenium $F(1,21) = 17.26$, $p = 0.0004$; D: selenium $F(1,20) = 7.86$, $p = 0.011$). A, B: Tukey test; C, D: Bonferroni’s test. *$p < 0.05$, **$p < 0.01$. Data are presented as mean ± SEM.
