# Supplementary material for: Effects of voluntary exercise, diet, and selenium on hypothalamic adult neurogenesis
Source: Stem Cell Reports. 2026 Jun 11;21(7):102953. doi: 10.1016/j.stemcr.2026.102953 (PMC13385432; doi:10.1016/j.stemcr.2026.102953)
Supplement: Document S2. Article plus supplemental information [file mmc2.pdf]

STEM CELL REPORTS
Article
ISSCR
OPEN ACCESS

# Effects of voluntary exercise, diet, and selenium on hypothalamic adult neurogenesis

Sara K.M. Jörgensen,[1] Rachel E. Martin,[1] Andrew Want,[2] James Morgan,[2] and David Petrik[1,3,*]
[1]School of Biosciences, Cardiff University, Cardiff CF10 3AX, UK
[2]School of Optometry and Vision Sciences, Cardiff University, Cardiff CF24 4HQ, UK
[3]Lead contact
*Correspondence: petrikd@cardiff.ac.uk
https://doi.org/10.1016/j.stemcr.2026.102953

## SUMMARY

Hypothalamic adult neurogenesis is implicated in energy homeostasis; however, the impact of physical exercise, particularly under high-fat diet (HFD) conditions, on this process remains unclear. To address this, we subjected mice to short-term and long-term voluntary running under control and HFD conditions. We show that long-term, but not short-term, running upregulates hypothalamic neurogenesis in Control mice. Conversely, short-term running rescues HFD-induced neurogenesis in the median eminence (ME), promoting the differentiation and survival of newly generated neurons. This rescue effect is absent with long-term running. Furthermore, we show that selenium mimics the effects of short-term running on ME neurogenesis and increases the activation and proliferation of hypothalamic adult neural stem cells, suggesting its role as a hypothalamic exerkine. Our findings indicate that voluntary exercise differentially influences adult neurogenesis in the hypothalamus compared to the hippocampus, with its neurogenic effects being modulated by diet, exercise duration, and regional differences within hypothalamic compartments.

## INTRODUCTION

In the medial basal hypothalamus (MBH), tanycytes, radial-glia-like cells line the third brain ventricle (3V) to form the hypothalamic ventricular zone (HVZ), the hypothalamic neurogenic niche (Xu et al., 2005). As adult hypothalamic neural stem cells (htNSCs) (Kokoeva et al., 2005; Lee et al., 2012), tanycytes regulate energy homeostasis and body weight (Li et al., 2012; McNay et al., 2012; Zhang et al., 2017). Both α-tanycytes and β-tanycytes (the latter in the median eminence (ME)) are neurogenic (Haan et al., 2013; Robins et al., 2013), generating either proopiomelanocortin-expressing (POMC+) or agouti-related peptide (AGRP+)/neuropeptide Y (NPY+) neurons, which suppress or promote eating, respectively (Kokoeva et al., 2005; Robins et al., 2013).

Hypothalamic adult neurogenesis (htAN) was shown to be influenced by exercise and diet. While short-term high-fat diet (HFD, lasting 1–2 weeks) increased MBH cell proliferation as a possible adaptative response (Gouaze et al., 2013; Jorgensen et al., 2024), long-term HFD (over 1 month) reduced survival of adult-generated hypothalamic neurons by increasing apoptosis (Jorgensen et al., 2024; Li et al., 2012; McNay et al., 2012). This suggests long-term HFD is detrimental to the differentiation and survival of adult-born hypothalamic neurons.

Exercise is well-known to upregulate adult neurogenesis in the hippocampal subgranular zone (SGZ), primarily by increasing cell proliferation within two weeks (Kronenberg et al., 2003). While the HVZ also responds to exercise, its effects on htNSC proliferation or new neuron survival under short- or long-term HFD remain unclear. Previous studies on exercise and htAN are limited. A 7-day forced running protocol increased new MBH cell survival, but only 0.5% were neurons (Borg et al., 2014). Conversely, long-term (8–12 weeks) voluntary exercise rescued HFD-induced decreases in MBH proliferation (Klein et al., 2019; Laing et al., 2016) and potentially increased new neuron proportion (Laing et al., 2016; Niwa et al., 2016).

However, these studies lacked crucial elements: phenotyping of proliferating cells or new neurons, lineage tracing, stereological quantification, or compartment-specific analysis, compromising their reliability and specificity. Thus, the effects of exercise and HFD on discrete htAN cellular stages, and their underlying mechanisms, require further rigorous investigation.

Exerkines, signaling molecules released in response to exercise, are prime mechanistic candidates (De Miguel et al., 2021). As an exerkine, selenium was recently found to be essential for exercise-induced hippocampal neurogenesis (Leiter et al., 2022). Antioxidant selenium transport protein SEPP1 and its receptor LRP8 mediate exercise-driven hippocampal NSC proliferation and activation (Leiter et al., 2022). In the hypothalamus, selenoproteins are highly expressed in AGRP+ and POMC+ neurons, regulating their redox status and reactive oxygen species signaling (Henry et al., 2015), indicating their importance for energy homeostasis. Indeed, unhealthy diets reduce hypothalamic selenoprotein expression (Schriever et al., 2017) and their loss-of-function leads to obesity (Zheng et al., 2020). Taken together, these findings suggest that selenium is critical for energy homeostasis. Despite this,


Published by Elsevier Inc. on behalf of International Society for Stem Cell Research.
This is an open access article under the CC BY license (http://creativecommons.org/licenses/by/4.0/).

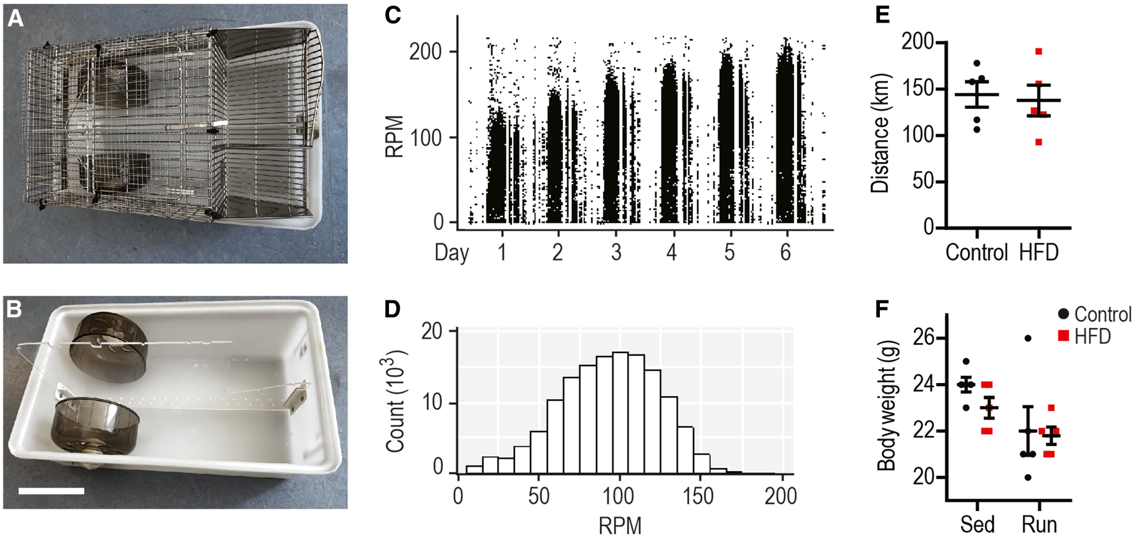


**Figure 1. Running in tandem housing cages reduces body weight in mice**

(A and B) Depiction of the tandem running cages with (A) or without (B) the cover mesh.

(C) An example of running wheel rotations per minute (RMP) over 6 days.

(D) Running wheel revolutions (counts) as a function of RPM over 14 days (14 days).

(E) Running distance over 14days by mice exposed to the control diet or HFD.

(F) 2-week running significantly reduces the body weight of mice on the control diet and HFD. $N = 5$/group. Scale bars (s.b.), 10 cm. Data information: $t$ test (E: not significant (n.s.)); two-Way ANOVA (F: exercise $F(1,16) = 6.65$, $p = 0.02$, diet $F(1,16) = 0.94$, $p = 0.35$, interaction $F(1,16) = 0.42$, $p = 0.53$). Data are presented as mean ± SEM.

selenium's role in htAN, particularly concerning exercise and diet, is unknown.

Here, we investigated the impact of voluntary exercise, combined with a control diet or HFD, on htNSCs and their progeny across different hypothalamic compartments using label-retention and lineage-tracing strategies. Our findings indicate that the htAN responds to long-term, but not short-term, exercise. Exercise can rescue htAN induced by short-term HFD, yet it fails to ameliorate the reduced survival of adult-born neurons caused by long-term HFD. Furthermore, our results suggest that selenium mimics some neurogenic effects of exercise. Collectively, our study demonstrates that exercise and diet influence the hypothalamic neurogenic niche differently compared to the hippocampal niche.

## RESULTS

### Running increases hippocampal adult neurogenesis in control diet and HFD

To assess the impact of voluntary exercise and diet on adult neurogenesis, we designed custom tandem-housing cages (Figures 1A and 1B), allowing individual activity monitoring (Figures 1C and 1D). Mice received either an *ad libitum* control diet or HFD (Jorgensen et al., 2024), which did not significantly alter running distance (Figure 1E). However, running significantly reduced body weight in both diet groups (Figure 1F, two-Way ANOVA). To confirm that voluntary exercise upregulates hippocampal adult neurogenesis as previously demonstrated (Kronenberg et al., 2003), we quantified cells in the SGZ for markers of cell proliferation (Ki67), immature neurons (doublecortin, DCX), and neural stem cells (glial fibrillary acidic protein, GFAP) (Figures S1A–S1D) (Jorgensen et al., 2024; Kempermann et al., 2004). Our results show that two weeks of running increased SGZ cell proliferation in both Control and HFD groups (Figures S1E and S1F), but only increased the proliferation of neuroblasts or NSCs in control (Figures S1G–S1J). These findings indicate that short-term running enhances hippocampal adult neurogenesis and validate our experimental approach.

### Short-term running rescues HFD-induced neurogenesis in the ME

Next, we determined how short-term (2-week) exercise and diet affected adult neurogenesis in the MBH. We used the BrdU "label retention" strategy to trace adult-generated cells (Xu et al., 2005). Confocal quantification of stained brain sections (Figures 2A–2I) revealed that in the ME, HFD increased the density of BrdU+DCX+ neurons but not BrdU+GFAP+ astrocytes (Figures 2J–2L). Importantly, the exercise reversed this HFD-induced increase in BrdU+ neurons.

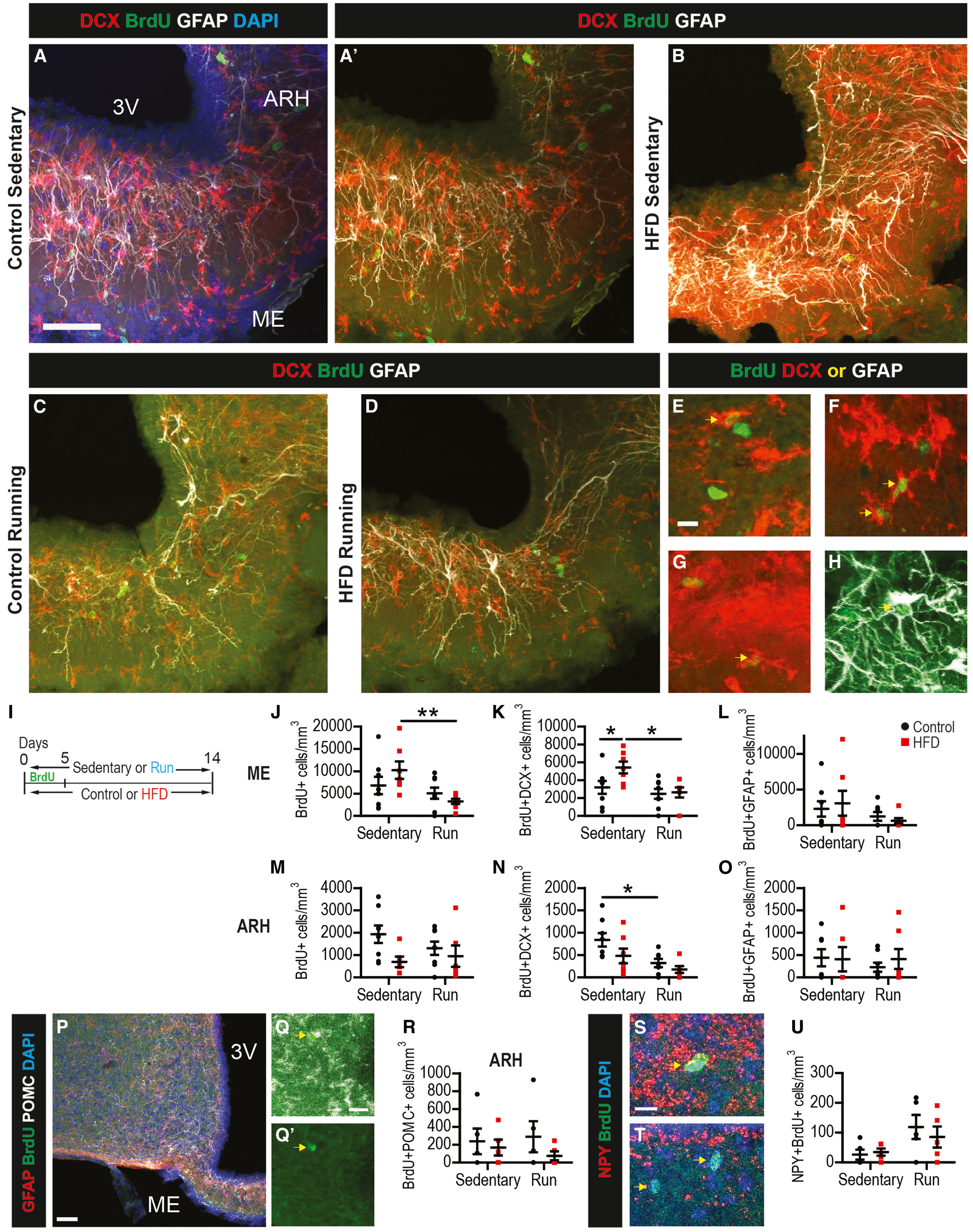


*(legend on next page)*

In the ARH, HFD reduced the density of BrdU+ cells, which exercise could not rescue (Figure 2M), whereas exercise reduced the number of BrdU+ neurons (Figure 2N) but had no effects on BrdU+ astrocytes (Figure 2O). This suggests a differential impact of these interventions across distinct MBH compartments. To further understand how these interventions influence htAN, we investigated the correlation between running distance and changes in cell density (Figures S2A–S2F). Linear regression showed no statistically significant correlation between BrdU+ cell or neuron density in the ME, ARH, or MBH for either Control or HFD runners. To ascertain if the interventions altered the phenotype of new, adult-generated neurons, we quantified the density of BrdU+ cells expressing the anorexigenic marker POMC (Figures 2P–2R) or the orexigenic marker NPY (Figures 2S–2U). Neither exercise nor HFD significantly changed the density of BrdU+POMC+ neurons, whereas exercise statistically significantly increased the density of BrdU+NPY+ cells. We also analyzed the neurites of BrdU+DCX+ cells (Figures S3A–S3H). In both the ME and ARH, HFD shortened primary branches; this effect was rescued by exercise in the ME but not in the ARH. Finally, we analyzed the density of activated caspase 3-positive (AC3+) cells in the parenchyma of the MBH (Figures S3I–S3K) and found no statistically significant difference between the treatment groups.

## HFD differently influences proliferation in the hypothalamic parenchyma and in the HVZ

The observed changes in immature neuron density within the ME could stem from altered hypothalamic cell proliferation. To address this, we quantified proliferating cells in the MBH from animals on the short-term protocol (Figures 3A–3E). In the ME, HFD significantly increased Ki67+ cell density (Figures 3F and 3G), suggesting elevated proliferation of cells. Conversely, the ARH exhibited no statistically significant change in cell proliferation across any group (Figures 3H and 3I). Changes in new neurons and cell proliferation in the ME might also reflect altered cellular dynamics of htNSCs. To address this, we quantified the number and density of htNSCs in the HVZ, identified by the tanycyte marker Retina and anterior neural fold homeobox transcription factor (RAX) (Figures 3J and 3K) (Miranda-Angulo et al., 2014; Yoo and Blackshaw, 2018). Running under the Control diet and HFD in sedentary mice both increased the number and density of RAX+ cells (Figures 3L and 3M), whereas HFD significantly reduced cell proliferation in the HVZ (Figure 3N). Collectively, these findings suggest the interventions may expand the htNSC pool.

## Long-term running increases hypothalamic neurogenesis, but cannot protect against the long-term HFD

To further elucidate the effects of exercise and diet on htAN, we used a three-month long-term protocol that combined the BrdU "label retention" strategy with lineage tracing of htNSCs in GRT transgenic reporter mice (Petrik et al., 2018). Tamoxifen administration in GRT mice induced recombination-dependent tdTomato (tdTom) expression in GLAST+ htNSCs and their progeny, allowing us to differentiate "'accumulative neurogenesis" (tdTom+ cells over 3 months) from "acute neurogenesis" (BrdU+ cells generated in the final 2 weeks of the protocol). Quantification of tdTom+ cells positive for the mature neuron marker NeuN+ (Figures 4A–4I) revealed that HFD dramatically reduced their density throughout the entire MBH, in the ARH, and in the MBH parenchyma, in both sedentary and running mice (Figures 4J–4L). In contrast, long-term running increased the density of NeuN+BrdU + neurons (Figure 4M) and BrdU+ cells in the MBH (Figure S4A). However, this neurogenic effect of running was absent in mice exposed to HFD (Figures 4N and 4O and Figures S4B–S4F). These results suggest that the long-term running

**Figure 2. Short-term running reverses aberrant HFD-induced hypothalamic neurogenesis**
(A–D) Representative confocal images of the MBH stained as indicated in control sedentary (A), HFD sedentary (B), control running (C), and HFD running mice (D).
(E–H) Representative confocal images of BrdU+DCX+ cells (E-G) and a BrdU+GFAP+ cell (H) in the ARH or ME.
(I) A schematic of the 2-week protocol.
Cell density in the ME (J–L) and in the ARH (M–O) of BrdU+ (J, M), BrdU+DCX+ (K, N), and BrdU+GFAP+ cells (L, O).
(P) MBH stained for GFAP, BrdU, and POMC.
(Q) A representative confocal image of a BrdU+POMC+ cell in the ARH.
(R) Quantification of the density of BrdU+POMC+ cells in the ARH.
(S and T) Representative confocal images of BrdU+NPY+ (S) and BrdU+NPY- (T) cells in the ARH and ME.
(U) Quantification of the density of BrdU+POMC+ cells in the MBH.
See also Figures S1 and S2. N = 6–8/group. S.b. = 50 μm (A-D, P), 20 μm (Q), and 10 μm (E-H, P). Data information: two-Way ANOVA (J: exercise $F(1,28) = 7.82$, $p = 0.0098$, diet and interaction: n.s.; K: exercise $F(1,28) = 7.41$, $p = 0.012$, diet and interaction: n.s.; L: all n.s.; M: diet $F(1,24) = 4.70$, $p = 0.040$, exercise and interaction: n.s.; N: exercise: $F(1,24) = 9.53$, $p = 0.005$, diet and interaction: n.s.; O, R: all n.s), one-way ANOVA (U: exercise $F(1,16) = 6.21$, $p = 0.024$). $*p < 0.05$ and $**p < 0.01$ (Bonferroni's test). Data are presented as mean ± SEM.

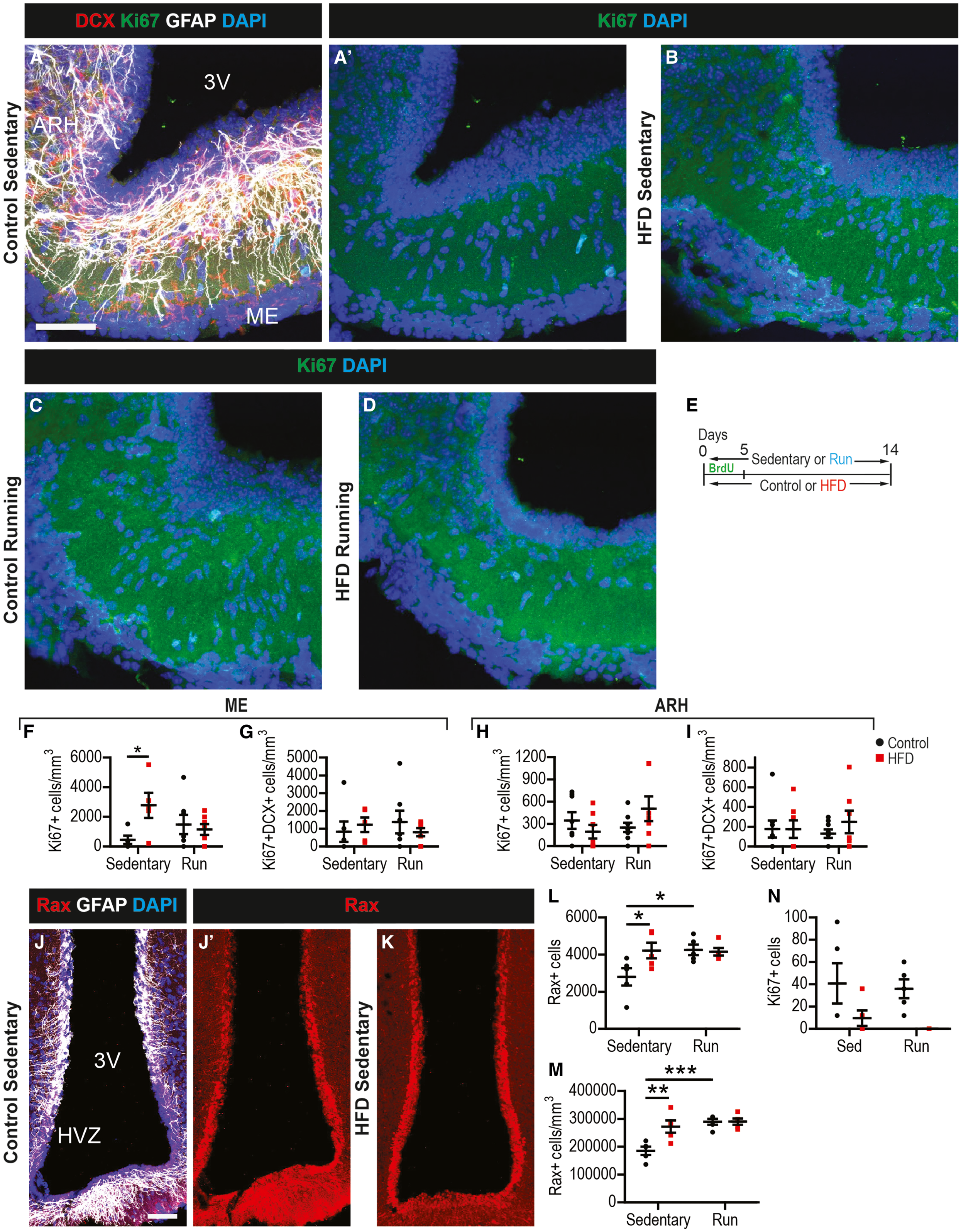


*(legend on next page)*

increases the acute neurogenesis in physiological conditions, but its neurogenic effects are blocked by HFD.

### Long-term running and HFD reduce the density of immature hypothalamic neurons

Our subsequent experiments investigated the effects of interventions on adult-generated immature neuron survival and cell proliferation within the MBH (Figure 5A). Cell quantification revealed that both long-term running under Control and HFD significantly reduced the density of adult-generated DCX+ cells. Two-Way ANOVA indicated that both interventions almost completely abolished tdTom+DCX+BrdU+ cells originating from GLAST+ htNSCs in the ARH and MBH parenchyma (Figures 5B–5H), but not in the MBH or ME (Figures S5A, S5B, and S5E). Conversely, long-term running increased the density of tdTom+BrdU + cells, though exclusively within the MBH parenchyma (Figure 5I) and not in other compartments (Figures S5C, S5F, and S5H). Notably, neither running nor HFD altered the density of immature neurons generated by acute neurogenesis in the final two weeks of the three-month protocol (Figures S5D, S5G, S5I, and S5J). To determine if these changes were linked to cell proliferation, we quantified Ki67+ cells in the MBH. Quantification showed that long-term HFD increased the density of Ki67+ cells in the ARH (Figures 5J and 5K); however, neither HFD nor running altered the density of Ki67+DCX+ or Ki67+tdTom+ cells in the MBH (Figures 5L and 5M). These results imply that the interventions do not significantly impact neurogenic proliferation.

### Running increases the expression of the selenoprotein receptor LRP8, and selenium increases the activation of htNSCs

Next, we investigated whether selenium could mimic exercise's effects on htAN. We first examined the hypothalamic expression of LRP8, a surface receptor for selenoprotein P (SEPP1), essential for exocrine effects of selenium in the adult hippocampus (Leiter et al., 2022). Our analysis showed almost all RAX+ cells in the HVZ express LRP8 (Figures 6A–6C) and two weeks of selenium-*l*-methionine exposure reduced the normalized pixel density of both LRP8 and RAX signals in the HVZ (Figures 6D and 6E). Importantly, two weeks of HFD decreased *Sepp1* expression (Figure 6F), whereas exercise increased *Lrp8* expression in the MBH (Figure 6G). These findings suggest that htNSCs express LRP8, and running upregulates its hypothalamic expression. To determine if selenium alters htNSCs and their proliferation in the context of diet or exercise, we quantified RAX+ and Ki67+ cells in the HVZ (Figure S6A). Two weeks of HFD and running, but not selenium, increased RAX+ cell numbers (Figures S6B and S6C). However, none of the interventions significantly altered Ki67+ cell numbers in the HVZ (Figures S6D and S6E). Because the HVZ exhibits low proliferation rates to properly assert stem cell dynamics, we further explored selenium's effects *in vitro*. Naive primary cultures of htNSCs were exposed to selenium-*l*-methionine and subjected to 4-day time-lapse imaging (Figures 6H and 6I). Individual cell clone analysis revealed a larger number and proportion of actively dividing clones in selenium-treated cells (Figures 6J and 6K), though no change in cells generated per clone (Figure 6L). In htNSC-derived neurospheres, selenium increased their size but not *Rax* or *Lrp8* expression (Figures 6M–6Q). Collectively, these results suggest selenium increases cell proliferation and activation of htNSCs but not their potency.

### Selenium rescues HFD-induced neurogenesis in the ME, mimicking the short-term running

To further explore the impact of selenium on htAN, we supplemented mice with selenium-*l*-methionine for two weeks, either with Control or HFD, and quantified new cell and neuron densities (Figures 7A and 7B). Selenium did not alter BrdU+ cell density in the ME or MBH (Figures 7C and S7A). However, in the ME, it significantly reduced BrdU+DCX+ cell density in HFD mice to levels similar to HFD runners (Figure 7D). A 3-way ANOVA revealed that diet and selenium significantly influenced BrdU+DCX+ cell density. Importantly, selenium also reduced BrdU+DCX+ cell density in Control mice both in the ME and MBH (Figures 7E, 7F, and S7B–S7D). These

**Figure 3. Changes in cell proliferation and htNSCs**

(A–D) Representative confocal images of the ARH and the ME stained as indicated in control sedentary (A), HFD sedentary (B), control running (C), and HFD running mice (D).

(E) A schematic of the 2-week protocol.

(F–I) Cell density in the ME (F-G) and in the ARH (H-I) of Ki67+ (F,H) and Ki67+DCX+ (G,I) cells.

(J–K) The MBH stained as indicated in control sedentary (J) and HFD sedentary (K).

(L–M) Quantification of the number (L) and density (M) of RAX+ cells in the HVZ.

(N) Quantification of Ki67+ cells in the HVZ.

See also Figures S2 and S3. $N = 5$/group. S.b. = 50 μm. Data information: two-Way ANOVA (F: interaction $F(1,21) = 5.27$, $p = 0.03$, G-I, L: all n.s.; M: exercise $F(1,16) = 15.90$, $p = 0.0011$, diet $F(1,16) = 8.12$, $p = 0.012$, interaction $F(1,16) = 7.85$, $p = 0.013$; N: exercise and interaction: n.s., diet $F(1,16) = 8.93$, $p = 0.009$). $^*p < 0.05$, $^{**}p < 0.01$, and $^{***}p < 0.001$ (Bonferroni's test). Data are presented as mean ± SEM.

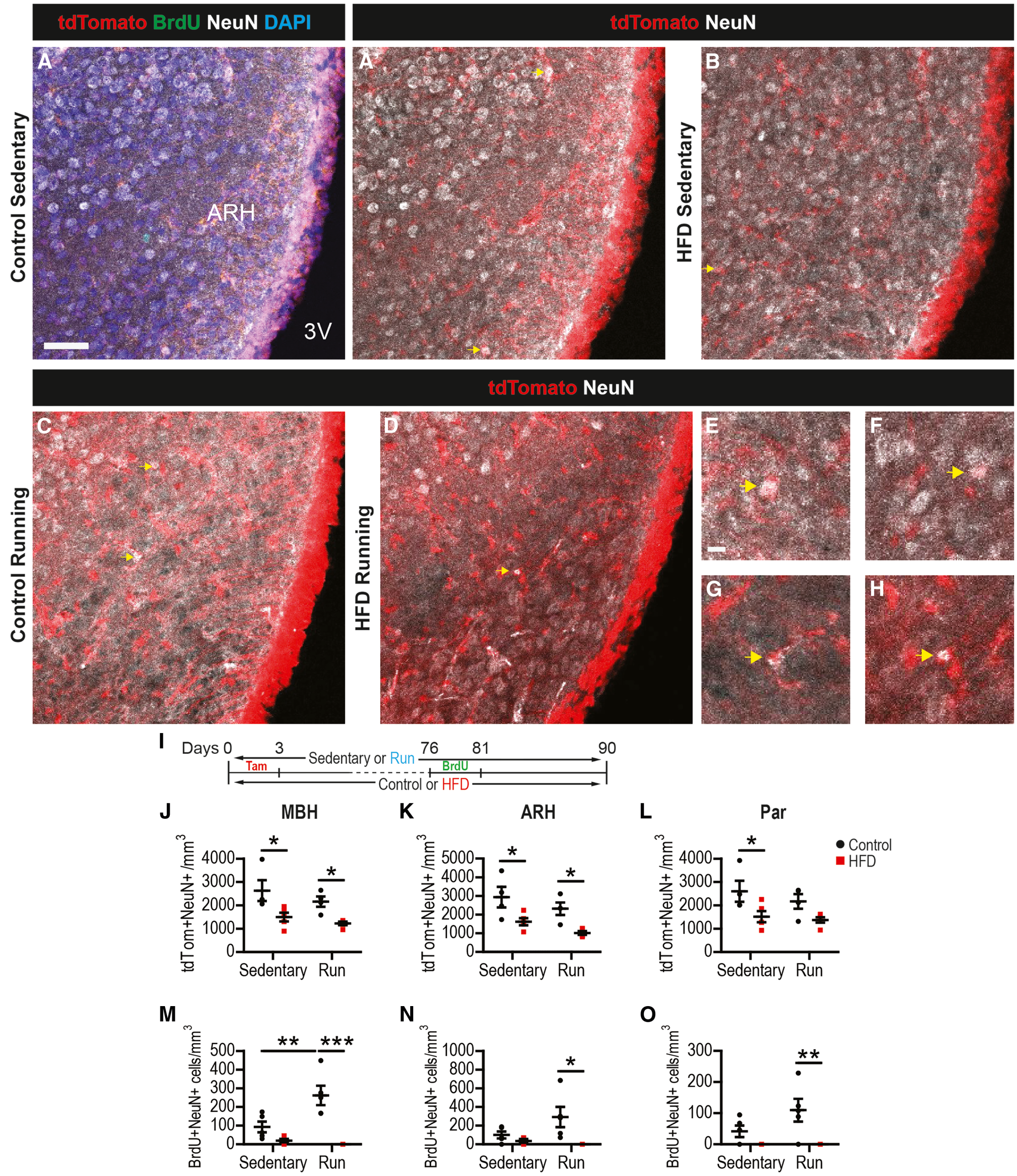


**Figure 4. Long-term running increases hypothalamic neurogenesis but does not protect against HFD**

(A–D) Representative confocal images of the ARH stained as indicated in control sedentary (A), HFD sedentary (B), control running (C), and HFD running mice (D).

(E–H) Representative confocal images of tdTom+NeuN + cells highlighted by arrows.

(I) A schematic of the 3-month protocol.

(J–O) Quantification of tdTom+NeuN+ (J-L) and BrdU+NeuN + cells (M-O) in the MBH (J,M), the ARH (K,N), and the parenchyma (L,O). See also Figure S4. N = 4–5/group. S.b. = 50 μm. Data information: two-Way ANOVA (J: exercise and interaction: n.s., diet $F(1,14) = 17.39$, $p = 0.0009$; K: exercise and interaction: n.s., diet $F(1,14) = 17.61$, $p = 0.0009$; L: exercise and interaction: n.s., diet $F(1,14) = 11.00$, $p = 0.0051$; M: exercise $F(1,14) = 4.85$, $p = 0.045$, diet $F(1,14) = 24.4$, $p = 0.0002$, interaction $F(1,14) = 7.77$, $p = 0.015$; N: exercise and interaction: n.s., diet $F(1,15) = 7.48$, $p = 0.016$; O: exercise and interaction: n.s., diet $F(1,15) = 11.96$, $p = 0.0035$). $*p < 0.05$, $**p < 0.01$, and $***p < 0.001$ (Bonferroni's test). Data are presented as mean ± SEM.

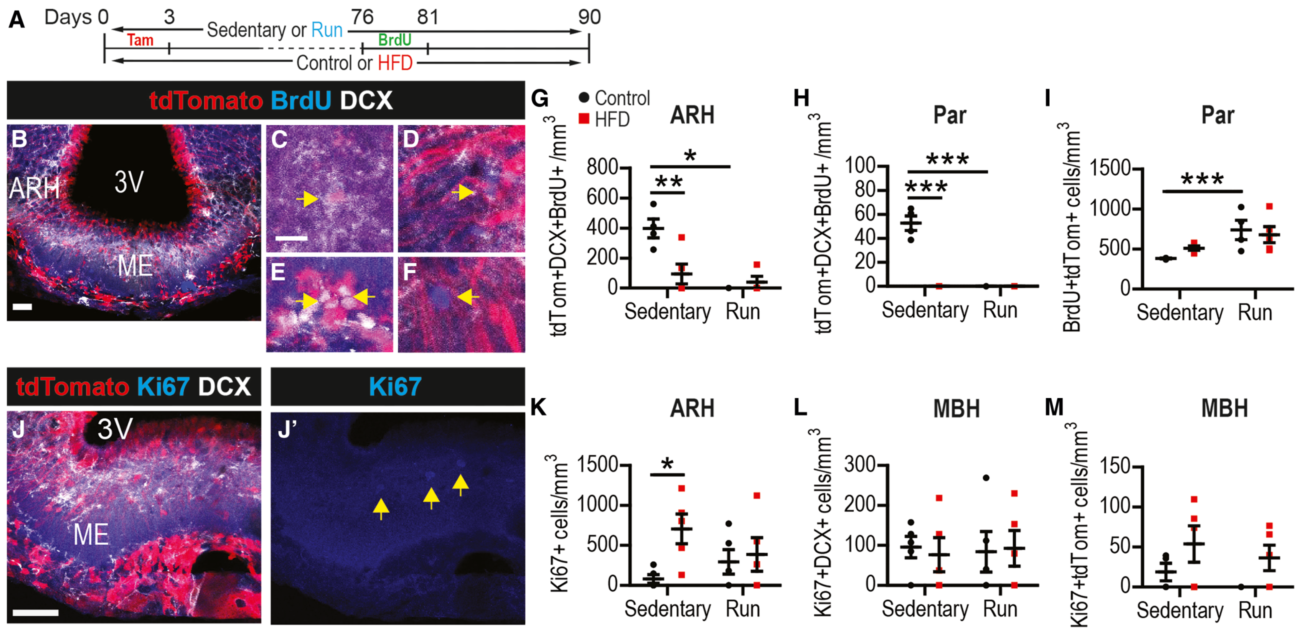


**Figure 5. Neither long-term running nor HFD affects neurogenic proliferation**

(A) A schematic of the 3-month protocol.

(B) A confocal image of the ARH and the ME stained as indicated.

(C–F) Representative confocal images of tdTom+DCX+BrdU+ (C,D), tdTom+DCX+ (E), and tdTom+BrdU+ (F) cells in the MBH.

(G–I) The density of tdTom+DCX+BrdU+ cells in the ARH (G) and the parenchymal density of tdTom+DCX+BrdU+ (H) and BrdU+tdTom+ cells (I).

(J) A confocal image of the ME stained as indicated, highlighting Ki67+ cells.

(K–M) The density of Ki67+ cells in the ARH (K) and the density in the MBH of Ki67+DCX+ (L) and Ki67+tdTom+ cells (M).

See also Figure S5. $N = 5$/group. S.b. = 20 μm (B-F), 50 μm (J). Data information: two-Way ANOVA (G: exercise $F(1,13) = 18.34$, $p = 0.0009$, diet $F(1,13) = 6.23$, $p = 0.027$, interaction $F(1,13) = 10.49$, $p = 0.0065$; H: all $F(1,11) = 62.81$, $p < 0.0001$; I: exercise $F(1,12) = 8.33$, $p = 0.014$, diet and interaction: n.s.; K: exercise and interaction: n.s., diet $(1,16) = 4.88$, $p = 0.042$; L and M: all n.s.). $^{*}p < 0.05$, $^{**}p < 0.01$, and $^{***}p < 0.001$ (Bonferroni's test). Data are presented as mean ± SEM.

results suggest that, under HFD conditions, selenium mimics the effects of running by rescuing the HFD-induced increase in adult-generated neurons in the ME. In contrast, selenium reduces new immature neuron survival under a control diet. The major effects of interventions are summarized in Figures 7G and 7H.

## DISCUSSION

Our study investigates the influence of exercise and diet on htAN across different hypothalamic compartments and at two distinct time points. Our custom-designed cages facilitate social interaction among tandem-housed animals while providing individual, monitored access to running wheels. This design mitigates social isolation stress, known to negatively affect adult neurogenesis (Stranahan et al., 2006). As shown previously (Gouaze et al., 2013; Niwa et al., 2016), the short-term HFD did not alter body weight, but the short-term exercise increased the number of proliferating cells in the SGZ in both Control and HFD. This corroborates a well-established paradigm of short-term exercise as a potent neurogenic factor in adult hippocampus (Kronenberg et al., 2003), thereby validating our experimental model.

In contrast to the SGZ, short-term running did not significantly alter cell proliferation or neurogenesis in the hypothalamus under control conditions. This finding differs from a previous study by (Borg et al., 2014), which reported increased hypothalamic proliferation with 7-day running but estimated that fewer than 0.5% of new cells were neurons. The discrepancy may stem from experimental design variations, such as a 6-day BrdU chase in Borg et al. and forced running, which can induce stress (Leasure and Jones, 2008) and affect cell dynamics. The differential responsiveness to running between hippocampal and hypothalamic neurogenic niches suggests a longer timescale is needed to stimulate the hypothalamus. Indeed, long-term running in our experiments increased "acute neurogenesis" under the control diet and may be influenced by

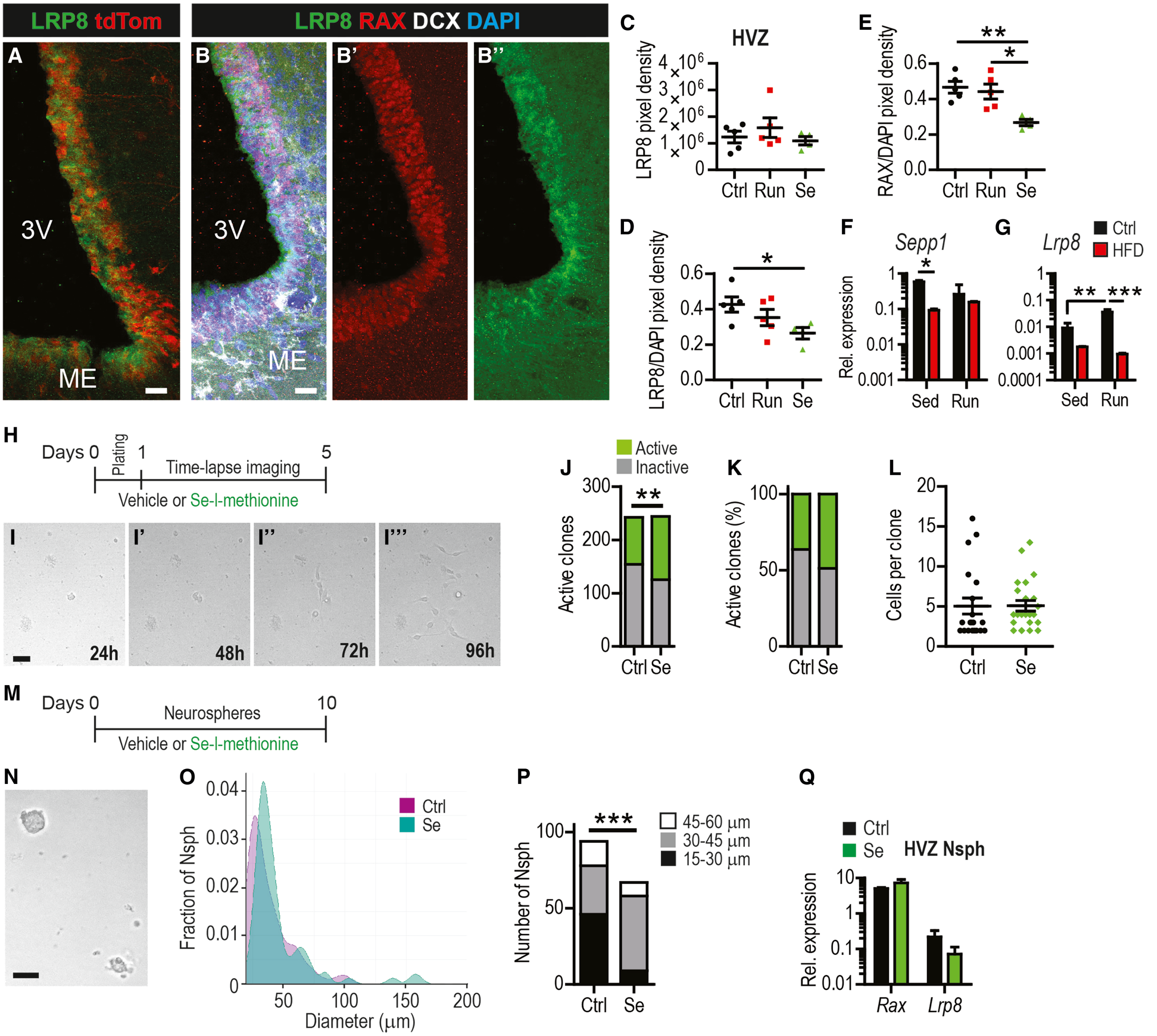


**Figure 6. Selenium activates the htNSCs**

(A and B) The HVZ and ME were stained for LRP8, RAX, and DCX.
(C–E) Quantification of the pixel density of LRP8 (C), LRP8/DAPI (D), and RAX/DAPI (E) in the HVZ.
(F–G) RT-qPCR expression of *Sepp1* (F) and *Lrp8* (G) in the MBH in Sedentary and Runner mice.
(H) A schematic of the time-lapse imaging experiment.
(I) A representative bright field microscope image of a single htNSC clone imaged over the period of 96 h (h).
(J and K) Quantification of the number (J) and the proportion (K) of active cell clones.
(L) Quantification of the number of cells generated per single cell clone.
(M) A schematic of the neurosphere protocol.
(N) A representative image of the htNSC-derived neurospheres.
(O) Kernel density plots of neurosphere diameter frequency distribution as a function of diameter.
(P) The number of neurospheres is shown binned by diameter.
(Q) RT-qPCR expression of *Rax* and *Lrp8* from htNSC-derived neurospheres treated with vehicle or selenium-*l*-methionine for 10 days.
See also Figure S6. N = 3–5/group. S.b. = 20 μm (A, B, I) 100 μm (M). Data information: *t* test ( L, Q: n.s., D: $p = 0.023$), One-Way ANOVA (C: n.s.; E: $F(2,13) = 8.96$, $p = 0.0049$), two-Way ANOVA (F: diet $F(1,8) = 6.71$, $p = 0.032$, interaction and exercise: n.s.; G: diet $F(1,9) = 24.08$, $p = 0.0008$, exercise $F(1,9) = 9.13$, $p = 0.014$, interaction $F(1,9) = 10.31$, $p = 0.011$), Fisher's Test (J, K: $p = 0.006$), chi-square (P: $p < 0.0001$), . *$p < 0.05$, **$p < 0.01$, and ***$p < 0.001$ (Bonferroni's test). Data are presented as mean ± SEM.

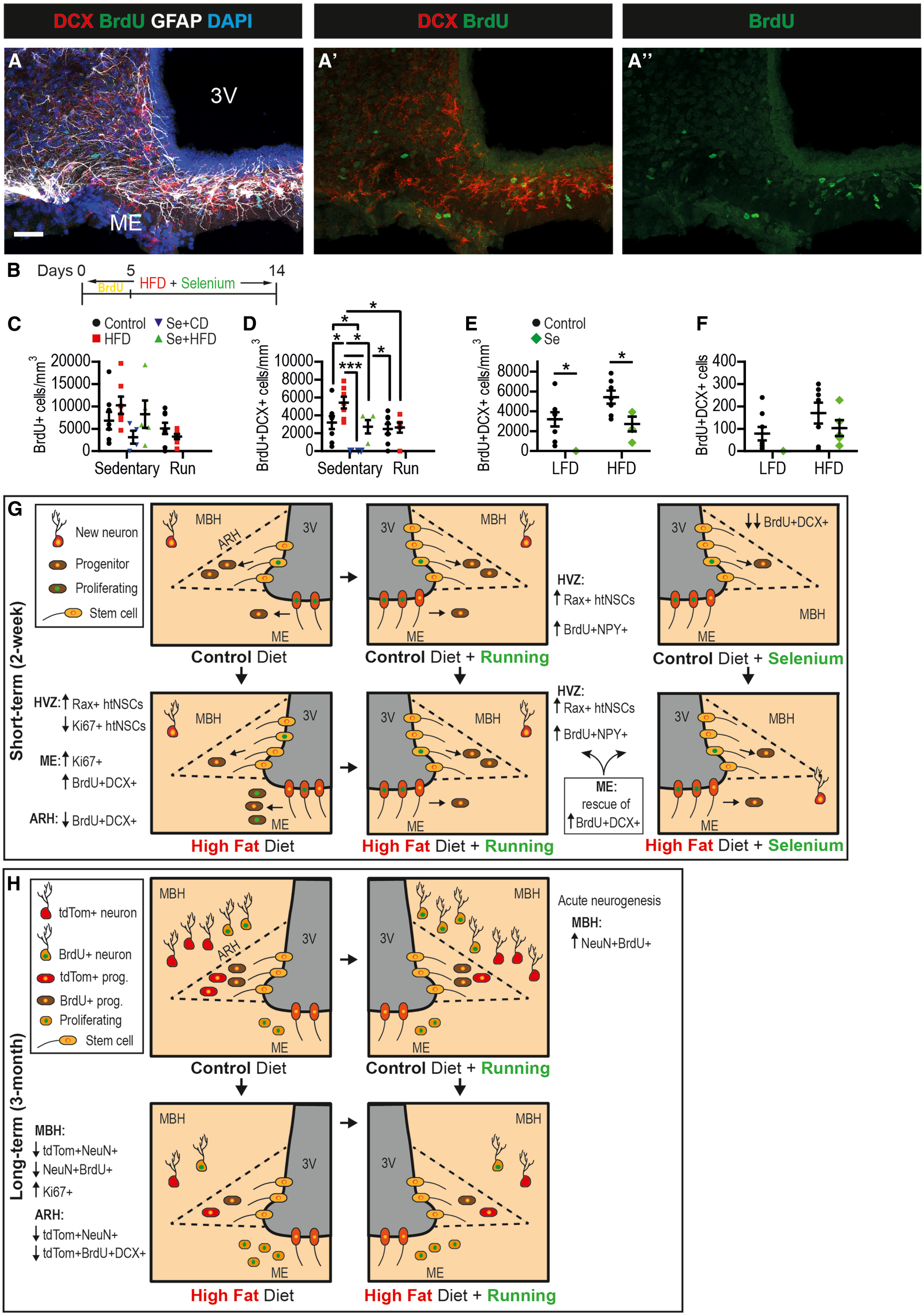


*(legend on next page)*

the slow proliferation of htNSCs (McNay et al., 2012), which have a significantly longer cell cycle than hippocampal NSCs (Jorgensen et al., 2024).

In contrast to running, which did not affect neurogenesis, short-term HFD increased neurogenesis in the ME, an effect reversed by running. This suggests two possibilities: either the increase is a compensatory response to HFD, rendered unnecessary by exercise as a protective intervention, or HFD induces aberrant neurogenesis that running rescues. Conversely, HFD reduced the density of new neurons in the ARH, an effect not mitigated by running. These findings highlight region-specific hypothalamic responses to interventions, corroborating previous observations of increased ME and reduced ARH neurogenesis by HFD (Lee et al., 2014). Notably, our study is the first to demonstrate that running reverses HFD-induced neurogenesis changes in the ME, likely due to its unique location outside the blood-brain barrier (BBB) (Lee et al., 2012, 2014). Indeed, the ME is more exposed to circulating factors than the BBB-protected ARH. In the ME, short-term HFDs disrupt tanycytes and the blood-CSF interface (Ramalho et al., 2018; Severi et al., 2021), whereas the ARH shows inflammation without early BBB leakage (Rijnsburger et al., 2019). In contrast, exercise reduces HFD-induced inflammation and microglial activation in the ARH (Sasaki et al., 2024), but its direct effects on the ME remain unknown. These differences in BBB protection and responsiveness likely underlie the region-specific effects on adult neurogenesis that we observed.

Interestingly, HFD decreased proliferation in the HVZ, yet it increased overall proliferation in the MBH parenchyma, further underscoring cell-specific effects of HFD on proliferation. Notably, none of the interventions altered $AC3^{+}$ cell numbers in the MBH, indicating that the observed neurogenesis changes were not affected by altered apoptosis. Taken together, these results suggest that short-term running influences neuronal differentiation or survival rather than cell proliferation in the ME.

Our observation that the short-term exercise increased the density of $BrdU^{+}NPY^{+}$ but not $BrdU^{+}POMC^{+}$ cells suggests that running promotes new orexigenic, but nor anorexigenic, neurons in the MBH, which is consistent with previous findings that exercise stimulates $NPY^{+}$ neurons (Landry et al., 2022). In contrast, HFD reduced the length of primary processes in $BrdU^{+}DCX^{+}$ cells in the ME and ARH, indicating for the first time that it may impair the dendritic development of new hypothalamic neurons.

In contrast to the short-term protocol, long-term running under Control diet conditions did not increase cumulative neurogenesis over 3 months in the MBH or ARH, corroborating findings of hypothalamic neurogenesis habituation to prolonged exercise (Klein et al., 2019). However, long-term running increased "acute" hypothalamic neurogenesis in the last two weeks. This differential response might stem from several factors. First, prolonged running could exhaust neurogenic htNSC and progenitor pools. Indeed, we observed reduced tdTomato+DCX+BrdU+ cell density yet no effect on Ki67+DCX+ cells, but we cannot exclude that running may temporarily increase GLAST+ htNSC neurogenesis at intermediate times. Second, our data do not fully address the influence of htNSC heterogeneity. Accumulative neurogenesis derived from GLAST+ htNSCs, while acute neurogenesis stemmed from any neurogenic htNSCs or progenitors unexhausted by terminal differentiation, including β-tanycytes (Robins et al., 2013). Indeed, we found tdTomato+ tanycytes also in the ventral aspects of the HVZ (in the infundibular recess), suggesting these were β1-tanycytes. But because these were rarely found in the most ventral ME, it may suggest that a subset of BrdU+ tdTomato-negative neurons generated during the acute neurogenesis may have originated from the GLAST-negative Nestin-positive β2-tanycytes located outside the BBB (Haan et al., 2013; Lee et al., 2012; Robins et al., 2013).

Our results demonstrate that long-term HFD is detrimental to hypothalamic neurogenesis, significantly reducing the density of new mature hypothalamic neurons generated by both accumulative and acute neurogenesis, an effect not rescued by running. Additionally, long-term HFD reduced cell density of tdTomato+DCX+BrdU+ but not DCX+BrdU + cells, suggesting reduced neuronal survival and differentiation from GLAST+ but not GLAST-negative

**Figure 7. Selenium supplementation reverses aberrant HFD-induced neurogenesis in the ME**

(A) A representative confocal image of ME and ARH stained as indicated.

(B) A schematic of the treatment protocol.

(C and D) Quantification of the density of BrdU+ (C) and BrdU+DCX+ cells (D) in the ME of sedentary and running animals exposed to control diet, HFD, and selenium.

(E and F) Quantification of the density (E) and the number (F) of BrdU+DCX+ cells in the ME in Control and HFD sedentary animals.

(G and H) A schematic summary of findings from 2-week (G) and 3-month (H) time points.

See also Figure S7. N = 4–8/group. S.b. = 50 μm. Data information: three-way ANOVA (C: exercise $F(1,33) = 4.60$, $p = 0.039$, diet-exercise interaction $F(1,33) = 3.87$, $p = 0.058$, diet and other interactions: n.s.; D: diet $F(1,31) = 8.33$, $p = 0.007$, selenium $F(1,31) = 17.12$, $p = 0.0003$, diet-exercise interaction $F(1,31) = 4.13$, $p = 0.051$, exercise and other interactions: n.s.), two-Way ANOVA (E: diet $F(1,19) = 11.30$, $p = 0.033$, selenium $F(1,19) = 16.0$, $p = 0.0008$, interaction: n.s.; F: diet $F(1,20) = 6.31$, $p = 0.021$, selenium and interaction: n.s.). $^{*}p < 0.05$, $^{**}p < 0.01$, and $^{***}p < 0.001$ (C, D: Tukey test; E, F: Bonferroni's test). Data are presented as mean ± SEM.

htNSCs and corroborating previous studies on the negative impact of long-term HFD on htAN (Jorgensen et al., 2024; Li et al., 2012; McNay et al., 2012). However, because we did not stereologically quantify the absolute number of all NeuN+ neurons in the MBH, our results do not show the proportional reduction of BrdU+NeuN + cells relative to total NeuN+ cells, which might also be reduced by long-term HFD.

There are previous studies that investigated the effects of long-term exercise on the hypothalamus, but their experimental paradigms make them incomparable with our results. For instance, Laing et al. (2016) found running could rescue HFD-induced POMC+ neuron reduction but did not differentiate between adult- and embryo-generated cells. Similarly, Niwa et al. (2016) showed a higher proportion of new hypothalamic neurons in the ARH and ME after 5–8 weeks of running in rats, but only in Control diet conditions. Conversely, Klein et al. used 3-month exercise and HFD, making their paradigm directly comparable with ours (Klein et al., 2019). Contrary to our findings, however, their study reported no HFD effect on new hypothalamic neuron numbers or subtypes compared to control or running, yet observed higher hypothalamic cell proliferation in HFD runners. Like our study, Klein et al. also noted a reduction in adult-generated cells due to running. However, key differences exist between the Klein et al. paradigms and ours: They used group-housed female mice, sedentary controls without locked running wheels, and a BrdU label-retention strategy for tracing 3-month-old cells. Thus, Klein et al. could only monitor survival, not differentiation, concluding that interventions do not alter long-term survival of early-generated cells.

Next, we investigated mechanisms by which exercise rescues HFD-induced increases in ME neurogenesis. Our study uniquely focused on selenium as a neurogenic exerkine in the adult hypothalamus. We found co-localization of RAX and LRP8 in HVZ cells, suggesting for the first time that tanycytes (as htNSCs) express *Lrp8*. Although neither *Sepp1* nor *Lrp8* are among the top 300 most expressed genes in tanycytes (Chen et al., 2017), various blood-brain interfaces express LRP8 (Vargas-Caraveo et al., 2017), and tanycytes do express Iodothyronine Deiodinase 2 (DIO2), a selenoprotein crucial for thyroid hormone synthesis, leading to the expectation that tanycytes express other selenoproteins or their receptors (Gong et al., 2018). Importantly, our results show that HFD decreases and exercise increases *Lrp8* expression in the MBH, which likely influences the effects of selenium supplementation on htAN.

To determine selenium's effects on htAN, we exposed mice or primary htNSCs *in vitro* to elevated selenium-L-methionine. We hypothesized that selenium supplementation would rescue HFD-induced ME neurogenesis, mimicking exercise. Indeed, selenium with HFD lowered ME BrdU+DCX+ cell density to levels observed in running plus HFD mice. Notably, this exercise-mimicking effect of selenium is specific to the ME and new adult-generated neurons, not affecting all cells. Although both exercise and selenium reduce the number of BrdU+DCX+ cells in the ME, it is possible that this effect is not beneficial if the HFD-increase in these cells is a compensatory response. In the control diet, selenium also significantly reduced the density of BrdU+DCX+ cells. This suggests that while selenium may be beneficial with an unhealthy diet, it could be detrimental to htAN under physiological conditions, consistent with selenium's beneficial effects being confined to a narrow concentration range (Genchi et al., 2023).

Next, we investigated whether selenium supplementation alters NSC proliferation and activation in the hypothalamus, as observed in the hippocampus (Leiter et al., 2022). Our results suggest that selenium increases the activation and proliferation of htNSCs and progenitors *in vitro*, which is not observed *in vivo*, possibly due to low proliferation dynamics in the HVZ. These results indicate that the rescue of HFD-induced ME neurogenesis by selenium is likely driven by differentiation, as selenium increased rather than decreased htNSC activation and proliferation.

Future experiments should focus on the long-term effects of selenium and on the molecular mechanisms of selenoproteins on hypothalamic neurogenesis and htNSCs. Given that SEPP1 is critical for hippocampal adult NSC activation, requiring metabolic changes (Knobloch et al., 2013; Leiter et al., 2022), selenium likely influences both redox and metabolic status of htNSCs. Future work should also establish causative links between exercise and selenoproteins in regulating htAN, further solidifying selenium as a major hypothalamic exerkine.

## RESOURCE AVAILABILITY

### Lead contact

Requests for further information and resources should be directed to and will be fulfilled by the lead contact, David Petrik (petrikd@cardiff.ac.uk).

### Materials availability

Non-commercial reagents or samples used in this study will be made available upon request to the lead contact, David Petrik, with a completed materials transfer agreement.

### Data and code availability

Any additional information required to reanalyze the data reported in this paper is available from the lead contact, David Petrik, upon request.

## ACKNOWLEDGMENTS

We thank Dr. Tara Walker (Queensland Brain Institute, Australia) for a very useful discussion about experimental paradigms for selenium. We thank Prof. Anne White (University of Manchester, UK)

for kindly sharing the anti-ACTH antibody developed in her laboratory. We also thank Caitlin Conway, Joseph Frullo, Grace Draper, and Charles De Almeida for their technical assistance. This work was supported by start-up funds from the Cardiff University School of Biosciences. SKMJ was supported by a PhD studentship from Cardiff University.

## AUTHOR CONTRIBUTIONS

S.K.M.J.: investigation. R.E.M.: investigation. A.W. and J.M.: resources. D.P.: conceptualization. resources. supervision. investigation. writing – original draft. writing—review and editing.

## DECLARATION OF INTERESTS

The authors declare no competing interests.

## STAR★METHODS

Detailed methods are provided in the online version of this paper and include the following:

- KEY RESOURCES TABLE
- EXPERIMENTAL MODEL
  - Mice
  - Mouse diets
  - Mouse running experiments
  - Selenium in mice
  - Cell cultures
- METHOD DETAILS
  - Brain tissue preparation and immunohistochemistry
  - Cell quantification
  - Neurite tracing
  - Time-lapse imaging
  - RT-qPCR
- QUANTIFICATION AND STATISTICAL ANALYSIS

## SUPPLEMENTAL INFORMATION

Supplemental information can be found online at https://doi.org/10.1016/j.stemcr.2026.102953.

Received: July 31, 2025
Revised: May 11, 2026
Accepted: May 12, 2026
Published: June 11, 2026

## REFERENCES

Bachmanov, A.A., Reed, D.R., Beauchamp, G.K., and Tordoff, M.G. (2002). Food intake, water intake, and drinking spout side preference of 28 mouse strains. Behav. Genet. *32*, 435–443. https://doi.org/10.1023/a:1020884312053.

Bankhead, P., Loughrey, M.B., Fernández, J.A., Dombrowski, Y., McArt, D.G., Dunne, P.D., McQuaid, S., Gray, R.T., Murray, L.J., Coleman, H.G., et al. (2017). QuPath: Open source software for digital pathology image analysis. Sci. Rep. *7*, 16878. https://doi.org/10.1038/s41598-017-17204-5.

Borg, M.L., Lemus, M., Reichenbach, A., Selathurai, A., Oldfield, B.J., Andrews, Z.B., and Watt, M.J. (2014). Hypothalamic neurogenesis is not required for the improved insulin sensitivity following exercise training. Diabetes *63*, 3647–3658. https://doi.org/10.2337/db13-1762.

Chen, R., Wu, X., Jiang, L., and Zhang, Y. (2017). Single-Cell RNA-Seq Reveals Hypothalamic Cell Diversity. Cell Rep. *18*, 3227–3241. https://doi.org/10.1016/j.celrep.2017.03.004.

De Miguel, Z., Khoury, N., Betley, M.J., Lehallier, B., Willoughby, D., Olsson, N., Yang, A.C., Hahn, O., Lu, N., Vest, R.T., et al. (2021). Exercise plasma boosts memory and dampens brain inflammation via clusterin. Nature *600*, 494–499. https://doi.org/10.1038/s41586-021-04183-x.

Genchi, G., Lauria, G., Catalano, A., Sinicropi, M.S., and Carocci, A. (2023). Biological Activity of Selenium and Its Impact on Human Health. Int. J. Mol. Sci. *24*, 2633. https://doi.org/10.3390/ijms24032633.

Gong, T., Torres, D.J., Berry, M.J., and Pitts, M.W. (2018). Hypothalamic redox balance and leptin signaling - Emerging role of selenoproteins. Free Radic. Biol. Med. *127*, 172–181. https://doi.org/10.1016/j.freeradbiomed.2018.02.038.

Gouazé, A., Brenachot, X., Rigault, C., Krezymon, A., Rauch, C., Nédélec, E., Lemoine, A., Gascuel, J., Bauer, S., Pénicaud, L., and Benani, A. (2013). Cerebral cell renewal in adult mice controls the onset of obesity. PLoS One *8*, e72029. https://doi.org/10.1371/journal.pone.0072029.

Gupta, B., Errington, A.C., Jimenez-Pascual, A., Eftychidis, V., Brabletz, S., Stemmler, M.P., Brabletz, T., Petrik, D., and Siebzehnrubl, F.A. (2021). The transcription factor ZEB1 regulates stem cell self-renewal and cell fate in the adult hippocampus. Cell Rep. *36*, 109588. https://doi.org/10.1016/j.celrep.2021.109588.

Haan, N., Goodman, T., Najdi-Samiei, A., Stratford, C.M., Rice, R., El Agha, E., Bellusci, S., and Hajihosseini, M.K. (2013). Fgf10-expressing tanycytes add new neurons to the appetite/energy-balance regulating centers of the postnatal and adult hypothalamus. J. Neurosci. *33*, 6170–6180. https://doi.org/10.1523/JNEUROSCI.2437-12.2013.

Henry, F.E., Sugino, K., Tozer, A., Branco, T., and Sternson, S.M. (2015). Cell type-specific transcriptomics of hypothalamic energy-sensing neuron responses to weight-loss. eLife *4*, e09800. https://doi.org/10.7554/eLife.09800.

Hilsenbeck, O., Schwarzfischer, M., Skylaki, S., Schauberger, B., Hoppe, P.S., Loeffler, D., Kokkaliaris, K.D., Hastreiter, S., Skylaki, E., Filipczyk, A., et al. (2016). Software tools for single-cell tracking and quantification of cellular and molecular properties. Nat. Biotechnol. *34*, 703–706. https://doi.org/10.1038/nbt.3626.

Jörgensen, S.K., Karnošová, A., Mazzaferro, S., Rowley, O., Chen, H.J.C., Robbins, S.J., Christofides, S., Merkle, F.T., Maletínská, L., and Petrik, D. (2023). An analogue of the Prolactin Releasing Peptide reduces obesity and promotes adult neurogenesis. EMBO Rep. *25*, 351–377. https://doi.org/10.1038/s44319-023-00016-2.

Kempermann, G., Jessberger, S., Steiner, B., and Kronenberg, G. (2004). Milestones of neuronal development in the adult hippocampus. Trends Neurosci. *27*, 447–452. https://doi.org/10.1016/j.tins.2004.05.013.

Klein, C., Jonas, W., Wiedmer, P., Schreyer, S., Akyüz, L., Spranger, J., Hellweg, R., and Steiner, B. (2019). High-fat Diet and Physical Exercise Differentially Modulate Adult Neurogenesis in the Mouse Hypothalamus. Neuroscience *400*, 146–156. https://doi.org/10.1016/j.neuroscience.2018.12.037.

Knobloch, M., Braun, S.M.G., Zurkirchen, L., von Schoultz, C., Zamboni, N., Araúzo-Bravo, M.J., Kovacs, W.J., Karalay, Ö., Suter, U., Machado, R.A.C., et al. (2013). Metabolic control of adult neural stem cell activity by Fasn-dependent lipogenesis. Nature *493*, 226–230. https://doi.org/10.1038/nature11689.

Kokoeva, M.V., Yin, H., and Flier, J.S. (2005). Neurogenesis in the hypothalamus of adult mice: potential role in energy balance. Science *310*, 679–683. https://doi.org/10.1126/science.1115360.

Kronenberg, G., Reuter, K., Steiner, B., Brandt, M.D., Jessberger, S., Yamaguchi, M., and Kempermann, G. (2003). Subpopulations of proliferating cells of the adult hippocampus respond differently to physiologic neurogenic stimuli. J. Comp. Neurol. *467*, 455–463. https://doi.org/10.1002/cne.10945.

Laing, B.T., Do, K., Matsubara, T., Wert, D.W., Avery, M.J., Langdon, E.M., Zheng, D., and Huang, H. (2016). Voluntary exercise improves hypothalamic and metabolic function in obese mice. J. Endocrinol. *229*, 109–122. https://doi.org/10.1530/JOE-15-0510.

Landry, T., Shookster, D., Chaves, A., Free, K., Nguyen, T., and Huang, H. (2022). Exercise increases NPY/AgRP and TH neuron activity in the hypothalamus of female mice. J. Endocrinol. *252*, 167–177. https://doi.org/10.1530/JOE-21-0250.

Leasure, J.L., and Jones, M. (2008). Forced and voluntary exercise differentially affect brain and behavior. Neuroscience *156*, 456–465. https://doi.org/10.1016/j.neuroscience.2008.07.041.

Lee, D.A., Bedont, J.L., Pak, T., Wang, H., Song, J., Miranda-Angulo, A., Takiar, V., Charubhumi, V., Balordi, F., Takebayashi, H., et al. (2012). Tanycytes of the hypothalamic median eminence form a diet-responsive neurogenic niche. Nat. Neurosci. *15*, 700–702. https://doi.org/10.1038/nn.3079.

Lee, D.A., Yoo, S., Pak, T., Salvatierra, J., Velarde, E., Aja, S., and Blackshaw, S. (2014). Dietary and sex-specific factors regulate hypothalamic neurogenesis in young adult mice. Front. Neurosci. *8*, 157. https://doi.org/10.3389/fnins.2014.00157.

Leiter, O., Zhuo, Z., Rust, R., Wasielewska, J.M., Grönnert, L., Kowal, S., Overall, R.W., Adusumilli, V.S., Blackmore, D.G., Southon, A., et al. (2022). Selenium mediates exercise-induced adult neurogenesis and reverses learning deficits induced by hippocampal injury and aging. Cell Metab. *34*, 408–423.e8. https://doi.org/10.1016/j.cmet.2022.01.005.

Li, J., Tang, Y., and Cai, D. (2012). IKKbeta/NF-kappaB disrupts adult hypothalamic neural stem cells to mediate a neurodegenerative mechanism of dietary obesity and pre-diabetes. Nat. Cell Biol. *14*, 999–1012. https://doi.org/10.1038/ncb2562.

Livak, K.J., and Schmittgen, T.D. (2001). Analysis of relative gene expression data using real-time quantitative PCR and the 2(-Delta Delta C(T)) Method. Methods *25*, 402–408. https://doi.org/10.1006/meth.2001.1262.

Madisen, L., Zwingman, T.A., Sunkin, S.M., Oh, S.W., Zariwala, H.A., Gu, H., Ng, L.L., Palmiter, R.D., Hawrylycz, M.J., Jones, A.R., et al. (2010). A robust and high-throughput Cre reporting and characterization system for the whole mouse brain. Nat. Neurosci. *13*, 133–140. https://doi.org/10.1038/nn.2467.

McNay, D.E.G., Briançon, N., Kokoeva, M.V., Maratos-Flier, E., and Flier, J.S. (2012). Remodeling of the arcuate nucleus energy-balance circuit is inhibited in obese mice. J. Clin. Investig. *122*, 142–152. https://doi.org/10.1172/JCI43134.

Miranda-Angulo, A.L., Byerly, M.S., Mesa, J., Wang, H., and Blackshaw, S. (2014). Rax regulates hypothalamic tanycyte differentiation and barrier function in mice. J. Comp. Neurol. *522*, 876–899. https://doi.org/10.1002/cne.23451.

Mori, T., Tanaka, K., Buffo, A., Wurst, W., Kühn, R., and Götz, M. (2006). Inducible gene deletion in astroglia and radial glia–a valuable tool for functional and lineage analysis. Glia *54*, 21–34. https://doi.org/10.1002/glia.20350.

Niwa, A., Nishibori, M., Hamasaki, S., Kobori, T., Liu, K., Wake, H., Mori, S., Yoshino, T., and Takahashi, H. (2016). Voluntary exercise induces neurogenesis in the hypothalamus and ependymal lining of the third ventricle. Brain Struct. Funct. *221*, 1653–1666. https://doi.org/10.1007/s00429-015-0995-x.

Petrik, D., Myoga, M.H., Grade, S., Gerkau, N.J., Pusch, M., Rose, C.R., Grothe, B., and Götz, M. (2018). Epithelial Sodium Channel Regulates Adult Neural Stem Cell Proliferation in a Flow-Dependent Manner. Cell Stem Cell *22*, 865–878.e8. https://doi.org/10.1016/j.stem.2018.04.016.

Ramalho, A.F., Bombassaro, B., Dragano, N.R., Solon, C., Morari, J., Fioravante, M., Barbizan, R., Velloso, L.A., and Araujo, E.P. (2018). Dietary fats promote functional and structural changes in the median eminence blood/spinal fluid interface-the protective role for BDNF. J. Neuroinflammation *15*, 10. https://doi.org/10.1186/s12974-017-1046-8.

Rijnsburger, M., Unmehopa, U.A., Eggels, L., Serlie, M.J., and la Fleur, S.E. (2019). One-week exposure to a free-choice high-fat high-sugar diet does not disrupt blood-brain barrier permeability in fed or overnight fasted rats. Nutr. Neurosci. *22*, 541–550. https://doi.org/10.1080/1028415X.2017.1418727.

Robins, S.C., Stewart, I., McNay, D.E., Taylor, V., Giachino, C., Goetz, M., Ninkovic, J., Briancon, N., Maratos-Flier, E., Flier, J.S., et al. (2013). alpha-Tanycytes of the adult hypothalamic third ventricle include distinct populations of FGF-responsive neural progenitors. Nat. Commun. *4*, 2049. https://doi.org/10.1038/ncomms3049.

Sasaki, T., Sugiyama, M., Kuno, M., Miyata, T., Kobayashi, T., Yasuda, Y., Onoue, T., Takagi, H., Hagiwara, D., Iwama, S., et al. (2024). Voluntary exercise suppresses inflammation and improves insulin resistance in the arcuate nucleus and ventral tegmental area in mice on a high-fat diet. Physiol. Behav. *287*, 114703. https://doi.org/10.1016/j.physbeh.2024.114703.

Schriever, S.C., Zimprich, A., Pfuhlmann, K., Baumann, P., Giesert, F., Klaus, V., Kabra, D.G., Hafen, U., Romanov, A., Tschöp, M.H., et al. (2017). Alterations in neuronal control of body weight and anxiety behavior by glutathione peroxidase 4 deficiency. Neuroscience *357*, 241–254. https://doi.org/10.1016/j.neuroscience.2017.05.050.

Severi, I., Fosca, M., Colleluori, G., Marini, F., Imperatori, L., Senzacqua, M., Di Vincenzo, A., Barbatelli, G., Fiori, F., Rau, J.V., and Giordano, A. (2021). High-Fat Diet Impairs Mouse Median Eminence: A Study by Transmission and Scanning Electron Microscopy Coupled with Raman Spectroscopy. Int. J. Mol. Sci. *22*, 8049. https://doi.org/10.3390/ijms22158049.

Stranahan, A.M., Khalil, D., and Gould, E. (2006). Social isolation delays the positive effects of running on adult neurogenesis. Nat. Neurosci. *9*, 526–533. https://doi.org/10.1038/nn1668.

Vargas-Caraveo, A., Sayd, A., Maus, S.R., Caso, J.R., Madrigal, J.L.M., García-Bueno, B., and Leza, J.C. (2017). Lipopolysaccharide enters the rat brain by a lipoprotein-mediated transport mechanism in physiological conditions. Sci. Rep. *7*, 13113. https://doi.org/10.1038/s41598-017-13302-6.

Xu, Y., Tamamaki, N., Noda, T., Kimura, K., Itokazu, Y., Matsumoto, N., Dezawa, M., and Ide, C. (2005). Neurogenesis in the ependymal layer of the adult rat 3rd ventricle. Exp. Neurol. *192*, 251–264. https://doi.org/10.1016/j.expneurol.2004.12.021.

Yoo, S., and Blackshaw, S. (2018). Regulation and function of neurogenesis in the adult mammalian hypothalamus. Prog. Neurobiol. *170*, 53–66. https://doi.org/10.1016/j.pneurobio.2018.04.001.

Zhang, Y., Kim, M.S., Jia, B., Yan, J., Zuniga-Hertz, J.P., Han, C., and Cai, D. (2017). Hypothalamic stem cells control ageing speed partly through exosomal miRNAs. Nature *548*, 52–57. https://doi.org/10.1038/nature23282.

Zheng, X., Ren, B., Li, X., Yan, H., Xie, Q., Liu, H., Zhou, J., Tian, J., and Huang, K. (2020). Selenoprotein F knockout leads to glucose and lipid metabolism disorders in mice. J. Biol. Inorg. Chem. *25*, 1009–1022. https://doi.org/10.1007/s00775-020-01821-z.

## STAR★METHODS

### KEY RESOURCES TABLE

| REAGENT or RESOURCE | SOURCE | IDENTIFIER |
|---|---|---|
| **Antibodies** | | |
| Rat monoclonal anti-BrdU | Bio-Rad | Cat# MCA6144; RRID: AB_3750324 |
| Mouse monoclonal anti-NeuN | Sigma-Aldrich | Cat# MAB377; RRID: AB_2298772 |
| Rabbit polyclonal anti-GFAP | Agilent | Cat# Z0334; RRID:AB_10013382 |
| Mouse monoclonal anti-HuC/HuD | Thermo Fisher | Cat# A-21271; RRID:AB_221448 |
| Chicken monoclonal anti-Ki67 | Encor | Cat# CPCA-Ki67; RRID: AB_2637049 |
| Rabbit polyclonal anti-cleaved caspase-3 | Cell Signaling | Cat# 9661; RRID:AB_2341188 |
| Mouse monoclonal doublecortin | Santa Cruz Biotechnology | Cat# sc-271390; RRID:AB_3073697 |
| Guinea pig polyclonal anti-RAX | Takara Bio | Cat# M229; RRID:AB_2783559 |
| Rabbit monoclonal anti-LRP8 | Thermo Fisher | Cat# MA5-53502; RRID:AB_3247973 |
| Rabbit polyclonal anti-Neuropeptide Y | Abcam | Cat# ab30914; RRID:AB_1566510 |
| Monoclonal antibody to ACTH (A1H5) | Anne White | RRID:AB_2756529 |
| **Chemicals, peptides, and recombinant proteins** | | |
| Optimal Cutting Temperature (OCT) Compound | Thermo Fisher | Cat# 6502 |
| DAPI ready made solution | Sigma-Aldrich | Cat# MBD0015 |
| Triton X-100 | Thermo Fisher | Cat# 13454259 |
| Prolong Diamond Antifade Mountant | Thermo Fisher | Cat# P36961 |
| Bovine Serum Albumin | Thermo Fisher | Cat# 12737119 |
| B27 supplement | Thermo Fisher | Cat# 17504044 |
| Penicillin-Streptomycin solution | Thermo Fisher | Cat# 15140122 |
| DMEM/F12 medium | Thermo Fisher | Cat# 11320033 |
| Hyaluronidase | Sigma-Aldrich | Cat# H3884 |
| Trypsin | Sigma-Aldrich | Cat# T9201 |
| Recombinant human EGF | Peprotech | Cat# AF-100-15 |
| Recombinant human FGF-2 | Peprotech | Cat# AF-100-18 |
| Poly-D-lysine | Sigma-Aldrich | Cat# P6407 |
| Tamoxifen | Sigma-Aldrich | Cat# T5648 |
| Corn oil | Sigma-Aldrich | Cat# C8267 |
| Formaldehyde | Sigma-Aldrich | Cat# 252549 |
| Seleno-L-methionine | Thermo Scientific | Cat# 15478449 |
| **Critical commercial assays** | | |

(*Continued on next page*)

*Continued*

| REAGENT or RESOURCE | SOURCE | IDENTIFIER |
|---|---|---|
| MyTaq HS Red Mix 2× | Bioline | Cat# BIO-25047 |
| MyTaq Extract-PCR kit | Bioline | Cat# BIO-21127 |
| RNeasy Plus Mini KIt | Qiagen | Cat# 74134 |
| SuperScript III Reverse Transcriptase Kit | Thermo Fisher | Cat# 18080044 |
| RNaseOUT Recombinant Ribonuclease Inhibitor | Thermo Fisher | Cat# 10777019 |
| Fast SYBR Green Master Mix | Thermo Fisher | Cat# 4385612 |
| **Experimental models: Organisms/strains** | | |
| Mouse: C57BL/6J | Charles River | 000664 |
| Mouse: GLAST::CreER$^{T2}$ | M. Götz, Munich | N/A |
| Mouse: Rosa26lox-stop-lox-tdTomato (Ai9) | O. Sansom, Glasgow | N/A |
| **Oligonucleotides** | | |
| Gapdh forward primer: TTCACCACCATGGAGAAGG | This paper | N/A |
| Gapdh reverse primer: CACACCCATCACAAACATGG | This paper | N/A |
| Lrp8 forward primer: AGATGGGCTCAACAGTCAAC | This paper | N/A |
| Lrp8 reverse primer: AGTGGGCGATCATAGTTGCT | This paper | N/A |
| Sepp1 forward primer: TGTTGAAGAAGCCATTAAGATCG | This paper | N/A |
| Sepp1 reverse primer: CACAGTTTTACAGAAGTCTTCATCTTC | This paper | N/A |
| Rax forward primer: CCGGAGTACGAAGCTACTAGG | This paper | N/A |
| Rax reverse primer: CATACCTGGACCCGAACCTC | This paper | N/A |
| **Software and algorithms** | | |
| ZEN microscopy software | Zeiss | https://www.zeiss.com/microscopy/en/products/software/zeiss-zen.html |
| ImageJ | HIH | https://imagej.net/ |
| GraphPad Prism | GraphPad Software, Inc. | https://www.graphpad.com/ |
| The Tracing Tool (tTt) software | Hilsenbeck et al. (2016) | https://bsse.ethz.ch/csd/software/ttt-and-qtfy.html |
| QuPath | Bankhead et al. (2017) | https://qupath.github.io |

## EXPERIMENTAL MODEL

### Mice

All UK experiments followed Animals (Scientific Procedures) Act 1986 (ASPA) guidelines and followed regulated procedures approved by UK Home Office and the Animal Welfare and Ethical Review Body of Cardiff University. Male mice were

group-housed (maximum 5/cage prior to housing in running cages) under 12h light/dark conditions with *ad libitum* food/water, weaned at 4 weeks, and 6–8 weeks old at study onset. C57BL/6J male mice were purchased from Charles River Laboratories at 5–6 weeks old. GLAST-CreER$^{T2}$ (Mori et al., 2006) and R26-tdTomato (Madisen et al., 2010) transgenic lines were maintained on a C57BL/6J background and crossed to generate GLAST-CreER$^{T2}$ x R26-tdTomato (GRT) male mice (Petrik et al., 2018) for experiments. Genotyping was performed using MyTaq Extract-PCR Kit (Bioline) and published primers (Gupta et al., 2021).

### Mouse diets

Mice were given either Control diet or High-Fat Diet (HFD). The Control diet (Low-Fat Diet, LFD) had following energy composition (% of calories): 24% from proteins, 7% from fats, and 67% from carbohydrates (Ssniff Spezialdiäten GmbH, Soest, Germany; #V1535 RM-Haltung, 15 mm pellets). The HFD was prepared from base Diet (Ssniff R/M-H #V1530-000 RM-Haltung, Mehl (ground/powder), Ssniff) as described previously (Jorgensen et al., 2024) and had energy content of 5.3 kcal/g (% of calories): 13% from proteins, 60% from fats, and 27% from carbohydrates. HFD pellets were stored at −80°C, thawed at 4°C two days before given to mice, and changed 2–3 times per week.

### Mouse running experiments

Mice were housed in custom-made cages equipped with a vertical running wheel and a wheel-counter. Mice were paired with a clear, perforated divider, enabling visual, auditory, and olfactory communication while ensuring individual, monitored access to running wheels. Sedentary controls were housed identically with a fixed wheel. For the short-term HFD/Running experiment (2 weeks), wild-type C56BL/6J male mice received either Control or HFD. Bromodeoxyuridine (BrdU; 1 mg/mL in 1% sucrose drinking water) was administered for the initial 5 days, followed by a 9-day BrdU chase (Jorgensen et al., 2024). Mouse weight was recorded at the start and end of the experiment. For the long-term HFD/Running experiment (3 months), male and female GRT mice received Control or HFD. At the experiment onset, mice were induced via 3 days of intraperitoneal (i.p.) Tamoxifen (dissolved in 10% ethanol and 90% corn oil) injections (150 mg/kg of body weight) administered every other day over the period of 5 days (Gupta et al., 2021) before being placed in running or sedentary cages. BrdU (as above) was administered during the first 5 days of the last two weeks (2.5 months after experiment start, 14 days before cull), followed by a 9-day chase. Mouse weight was recorded at the start, end, and biweekly throughout the 3-month period. Running distance was calculated from wheel revolutions of a known diameter (13 cm).

### Selenium in mice

The diets provided to mice (Ssniff #V1535 and #V1530) contain 0.3 mg of selenium per 1 kg. WT C56BL/6j male mice were administered 50 μM selenium-*l*-methionine (CAS 3211-76-5; 98% purity) in drinking water for 2 weeks as described before (Leiter et al., 2022). Mice were housed in the sedentary cages and exposed to Control or HFD and BrdU in drinking water (5 days, 9-day chase as above). Selenium intake for experimental mice was estimated based on reported average daily food (~4 g) and water (~6 mL) consumption in adult C57BL/6J mice (Bachmanov et al., 2002). The estimated baseline selenium intake was ~1.2 μg/day/animal. The selenium supplementation increased the estimated daily selenium intake to ~24 μg/day/animal, representing a 20-fold increase relative to baseline.

### Cell cultures

Primary cell cultures were prepared from dissected 3V walls of adult C57BL/6J male mice. Cells were cultured as adherent cultures (20,000 cells/0.5 mL medium on PDL-coated wells) or neurospheres in proliferating conditions (with EGF and FGF-2, both 10 ng/mL) as previously described (Jorgensen et al., 2024). Naive cells were treated with 50 μM selenium-L-methionine or vehicle (water) for 4 days (adherent cultures) or 10 days (neurospheres). Neurosphere absolute numbers were quantified by exhaustive counting per well, and their diameters were measured using ImageJ.

## METHOD DETAILS

### Brain tissue preparation and immunohistochemistry

Euthanal-anaesthetized mice were transcardially perfused with ice-cold PBS followed by perfusion with ice-cold 4% paraformaldehyde. The brains were isolated and postfixed in 4% PFA for overnight. The PFA was replaced by 30% sucrose (with 0.1% $NaN_3$ in 1xPBS) and brains let to sink at room temperature. 50 μm coronal sections (bregma −0.6 to −4.0 mm) were cut on a cryostat in serial sets of 12 for stereological evaluation. Slide-mounted immunohistochemistry (IHC) was

performed, using following dilutions of primary antibodies: anti-BrdU (1:400), anti-NeuN (1:500), anti-GFAP (1:500), anti-HuC/HuD (1:500), anti-Ki67 (1:400), anti-activated caspase 3 (AC3, 1:500), anti-DCX (1:300), anti-RAX (1:200), anti-LRP8 (1:600), anti-Neuropeptide Y (NPY, 1:500), anti-ACTH to stain for POMC (1:5000). Two antigen retrieval strategies were used. For DCX or Ki67 staining, the pretreatment with 10mM tri-sodium citrate (pH = 6.0, 0.03% Triton X-100) in 70°C (15 min) was used. For BrdU IHC, a pretreatment with 2.5N hydrochloric acid (30 min) at room temperature (RT) was followed by a neutralisation with 0.1 M sodium borate ($Na_2B_4O_7$, pH = 8.5, 10 min) at RT. Blocking with the carrier (1× PBS, 0.5% Triton X-100, 5% normal donkey serum or 2% bovine serum albumin, minimum 20 min) was followed by incubation with the primary antibodies at RT overnight. After three washes in 1× PBS, the secondary antibodies were incubated for 1.5–2 hours at RT. All secondary antibodies were Alexa-conjugated antibodies (1:300, Thermo Fisher). Sections were counterstained with DAPI ready-made (1:1000). Slides were mounted and cover-slipped using ProLong Diamond antifade mountant.

### Cell quantification

Stereological quantification of marker-positive cells in the Medial Basal Hypothalamus (MBH) and the hippocampal Subgranular Zone (SGZ) was performed as previously described (Jorgensen et al., 2024). Coronal sections (1 in 12 series) containing the hippocampus (bregma −0.6 to −4.0 mm) and MBH (bregma −1.2 to −2.3 mm) were stained. Note that tanycytes are located between bregma −1.3 to −2.5 mm. z stack images were acquired using a Zeiss Observer.Z1 LSM780 confocal microscope with 20× or 40× apochromatic objectives, controlled by ZEN Blue software (Zeiss). Imaging was performed by an observer blinded to brain genotype. To quantify the absolute number of marker-positive cells per tissue volume, all cells positive for one or more antigens were counted within defined Regions of Interest (ROIs). For the MBH, ROIs included discrete anatomical structures: the MBH parenchyma (Arcuate Nucleus (ARH), Dorsomedial Nucleus (DMN), Ventromedial Nucleus (VMN)), and the ME. In the hippocampus, the ROI was restricted to the SGZ. ROI area was determined as previously described to calculate MBH, ME, Arc, or HVZ volumes (Jorgensen et al., 2024). RAX+ tanycyte numbers and HVZ area were automatically quantified per brain section by user-trained software QuPath (Bankhead et al., 2017). A background ratio of 30 pixels minimized noise, while maximum/minimum cell areas, manually determined from samples of smallest and largest RAX+ cells, prevented artifact detection. Images were smoothed (sigma = 1.5 pixels, threshold = 20) for optimal cell detection. z stack images were acquired using a Zeiss Observer.Z1 LSM780 confocal microscope using a 40× objective lens, controlled by ZEN Black software (Zeiss). Imaging and analysis were performed by an observer blind to the animal conditions. Using ZEN Blue software (Zeiss), BrdU+ cells were also analyzed for the overlap with NPY signal. BrdU+NPY+ cells were defined by either the overlap of these two signals or as BrdU+ nuclei surrounded by NPY+ processes.

### Neurite tracing

Using the Simple Neurite Tracer (SNT) plugin in ImageJ, we traced the cellular processes of $BrdU^+DCX^+$ cells in the ME and ARH using orthogonal projections of confocal z stack images acquired with a Zeiss LSM780 system (see above). The total length of primary branches per cell and the average branch length were then analyzed.

### Time-lapse imaging

Primary cell cultures were continuously imaged at 37°C using a Zeiss Axio Observer 7 microscope with Zeiss Definitive Focus module, as previously described (Jorgensen et al., 2024). Following imaging, cells were stained for RAX and GFAP. Cell cycle length, number of cell divisions, and cells per clone were determined using The Tracking Tool (Hilsenbeck et al., 2016). The proportion of actively dividing cells was derived from time-lapse video analysis in ZEN Blue (Zeiss), also as previously described (Jorgensen et al., 2024).

### RT-qPCR

Total RNA was isolated from the MBH tissue or HVZ-derived neurospheres using the RNeasy Mini Plus Kit. MBH tissue was sampled via 1 mm rapid biopsy punch (WPI, Hitchin, UK) from acutely isolated, live 300 μm coronal brain sections maintained in DMEM/F12 medium. Flash-frozen hypothalamic punches and neurospheres were thawed and triturated for cell disruption. RNA was reverse transcribed into cDNA using SuperScript III polymerase with random primers and RNaseOUT. qPCR was performed in technical duplicates using Fast SYBR Green dye on a StepOne Plus Real-Time PCR system (Life Technologies). Cycling conditions were: 95°C for 5 min, followed by 40 cycles of 95°C denaturation and 60°C annealing/elongation. Results were analyzed using AccuSEQ software (Life Technologies), with amplification cycle (Ct) values determined by the maximum 2nd derivative method and quantified using the $2^{-\Delta\Delta Ct}$ method (Livak and Schmittgen,

2001) normalized to *Gapdh*. Primer sequences (250 nM final concentration) were designed by the Primer-BLAST online tool (NCBI-NIH).

## QUANTIFICATION AND STATISTICAL ANALYSIS

Replicate numbers are detailed in Figure legends. Data were analyzed using Microsoft Excel and GraphPad Prism, following a specific statistical algorithm. Outliers were identified and removed via Grubb's test (ESD method, $\alpha = 0.05$). Normality was assessed by D'Agostino & Pearson's omnibus test (or Kolmogorov–Smirnov for $N < 5$). Normally distributed data underwent parametric tests. Simple comparisons of two groups used the un-paired two-tailed *t* test. For multiple factor/group comparisons, One-way, two-way, or three-way ANOVA was applied, followed by Bonferroni's or Tukey post-hoc tests. Contingency distributions were tested with Chi-square or Fisher's exact tests. Data are presented as mean ± standard error of mean (SEM). Results were considered significant at $p < 0.05$ (*), with $p < 0.01$ (**) and $p < 0.001$ (***).

**Stem Cell Reports, Volume *21***

## Supplemental Information

# Effects of voluntary exercise, diet, and selenium on hypothalamic adult neurogenesis

**Sara K.M. Jörgensen, Rachel E. Martin, Andrew Want, James Morgan, and David Petrik**

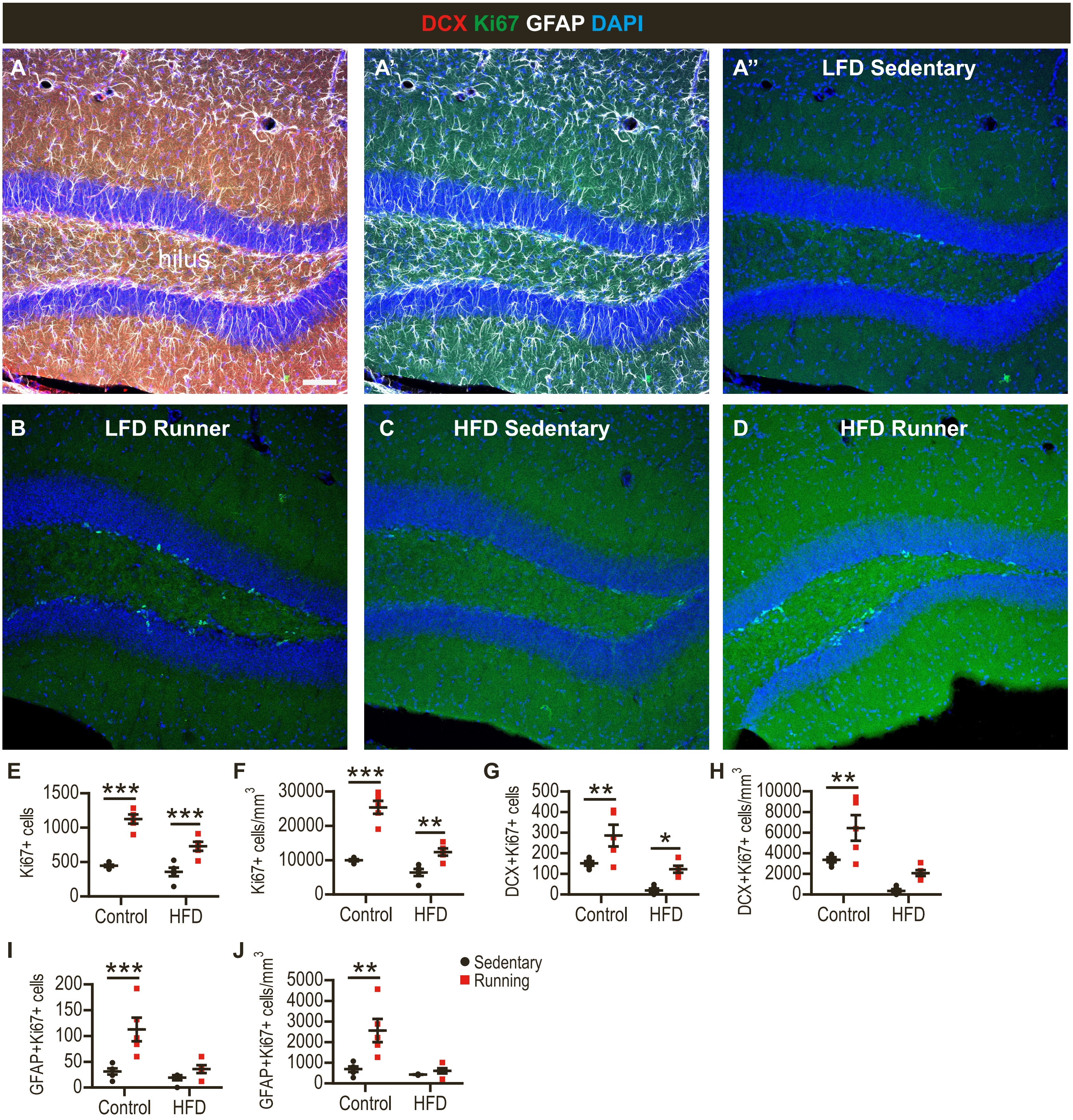


**Figure S1. Running increases cell proliferation in the SGZ in both Control and HFD, related to Figure 2**

(A-D) Representative confocal images of SGZ stained for DCX, Ki67 and GFAP in Control Sedentary (A), Control Runner (B), HFD Sedentary (C), and HFD Runner (D).

(E-J) Quantification of absolute number and density of proliferating cells (E-F), proliferating neuroblasts (G-H), and proliferating neural stem cells (I-J) in the SGZ.

N = 5/group. Scale bar: 50 μm. Data information: Two-Way ANOVA (panel E: exercise $F(1,16) = 85.87$, $p < 0.0001$, diet $F(1,16) = 18.27$, $p = 0.0006$, interaction $F(1,16) = 7.33$, $p < 0.016$; F: exercise $F(1,16) = 78.35$, $p < 0.0001$, diet $F(1,16) = 47.40$, $p < 0.0001$, interaction $F(1,16) = 15.38$, $p = 0.0012$; G: exercise $F(1,16) = 17.19$, $p = 0.0008$, diet $F(1,16) = 26.54$, $p < 0.0001$, interaction $F(1,16) = 0.3$, $p = 0.59$; H: exercise $F(1,16) = 13.4$, $p = 0.0021$, diet $F(1,16) = 31.81$, $p < 0.0001$, interaction $F(1,16) = 1.05$, $p = 0.32$; I: exercise $F(1,16) = 15.01$, $p = 0.0013$, diet $F(1,16) = 12.22$, $p = 0.0.003$, interaction $F(1,16) = 6.51$, $p = 0.02$; J: exercise $F(1,15) = 22.78$, $p = 0.0056$, diet $F(1,15) = 26.74$, $p =0.0032$, interaction $F(1,15) = 15.62$, $p = 0.017$).

$^{**}p < 0.01$, $^{***}p < 0.001$ (Bonferroni's test). Data are presented as mean ± SEM.

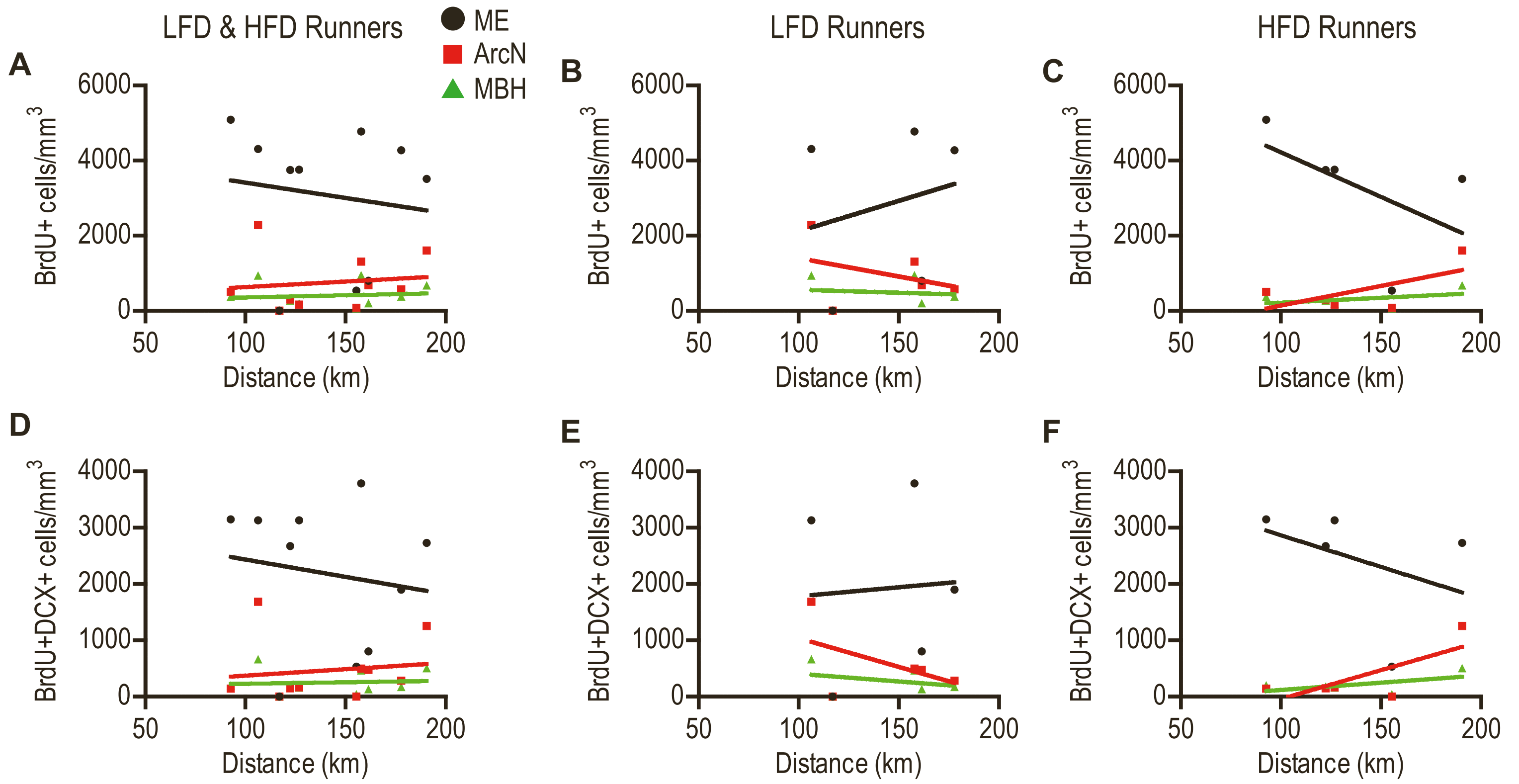


**Figure S2. No correlation between running distance and density of cells in different MBH compartments, related to Figure 2 and 3**

(A-C) Density of BrdU+ cells as a function of distance ran (kilometres in 14 days) in Control (LFD) and HFD animals combined (A), in Control Runners (B) and in HFD Runners (C).

(D-F) Density of BrdU+DCX+ cells as a function of distance ran (kilometres in 14 days) in Control (LFD) and HFD animals combined (D), in Control Runners (E) and in HFD Runners (F).

Individual points depict the cell density, and the corresponding running distance for each individual animal.

In all graphs, data are depicted for the cell densities in the ME (black circles), the ARH (red squares) and the MBH (green triangles).

Data information: Linear regression (all n.s.).

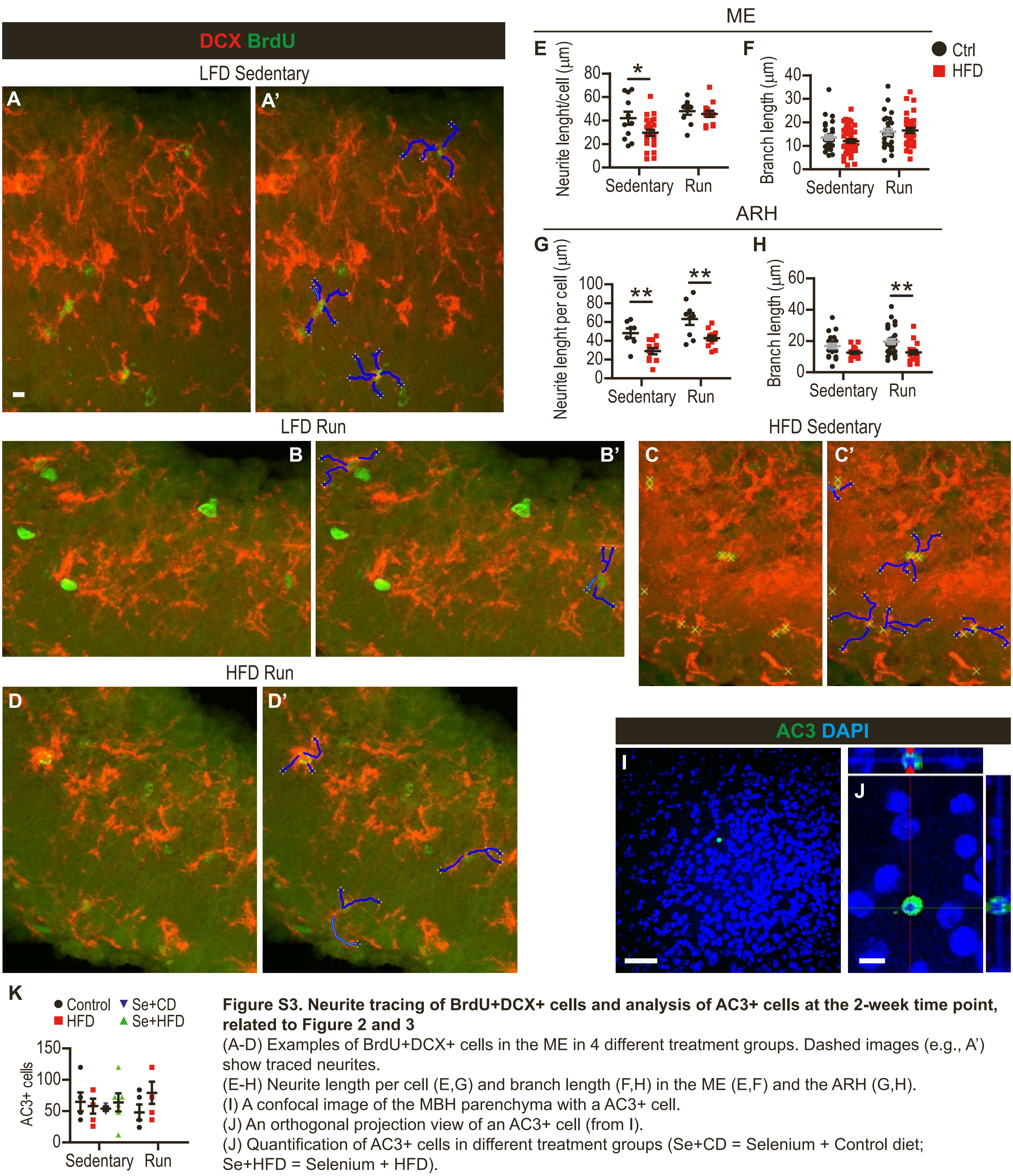


**Figure S3. Neurite tracing of BrdU+DCX+ cells and analysis of AC3+ cells at the 2-week time point, related to Figure 2 and 3**

(A-D) Examples of BrdU+DCX+ cells in the ME in 4 different treatment groups. Dashed images (e.g., A') show traced neurites.

(E-H) Neurite length per cell (E,G) and branch length (F,H) in the ME (E,F) and the ARH (G,H).

(I) A confocal image of the MBH parenchyma with a AC3+ cell.

(J) An orthogonal projection view of an AC3+ cell (from I).

(J) Quantification of AC3+ cells in different treatment groups (Se+CD = Selenium + Control diet; Se+HFD = Selenium + HFD).

N = 5/group. S.b. = 10 µm (A,J), 50 µm (I).

Data information: Two-Way ANOVA (panel E: exercise $F(1,54) = 9.28$, $p = 0.0036$, diet $F(1:54) = 4.14$, $p = 0.047$, interaction: n.s.; F: exercise $F(1,148) = 11.78$, $p = 0.0008$, diet & interaction: n.s.; G: exercise $F(1,36) = 11.30$, $p = 0.0019$, diet $F(1,36) = 21.03$, $p < 0.0001$, int.: n.s.; H: diet $F(1,85) = 14.41$, $p = 0.0003$, exercise & interaction: n.s), Three-Way ANOVA (panel K: all n.s.).

*$p < 0.05$, *$p < 0.01$ (Bonferroni's test). Data are presented as mean ± SEM.

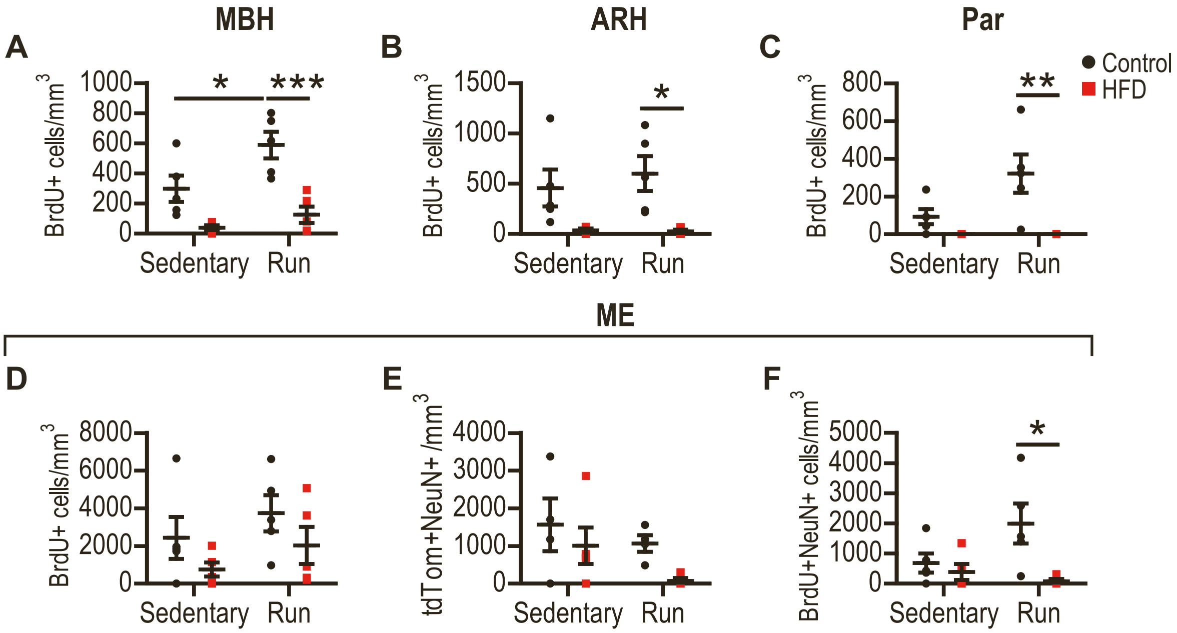


**Figure S4. Long-term HFD reduces the density of new cells, related to Figure 4**

(A-C) Density of BrdU+ in the MBH (A), the ARH (B), and parenchyma (C).

(D-F) Density of BrdU+ (D), tdTom+NeuN+ (E), and BrdU+NeuN+ (F) cells in the ME.

N = 5/group. Data information: Two-Way ANOVA (panel A: exercise $F(1,15) = 6.75$, $p = 0.02$, diet $F(1,15) = 24.93$, $p = 0.0002$, interaction: n.s.; B: exercise and interaction: n.s., diet $F(1,15) = 13.65$, $p = 0.0022$; C: exercise and interaction: n.s., diet $F(1,15) = 11.03$, $p = 0.0051$; D: all n.s.; E: all n.s.; F: exercise and interaction: n.s., diet $F(1,15) = 7.11$, $p = 0.018$).

*$p < 0.05$, ***$p < 0.001$ (Bonferroni's test). Data are presented as mean ± SEM.

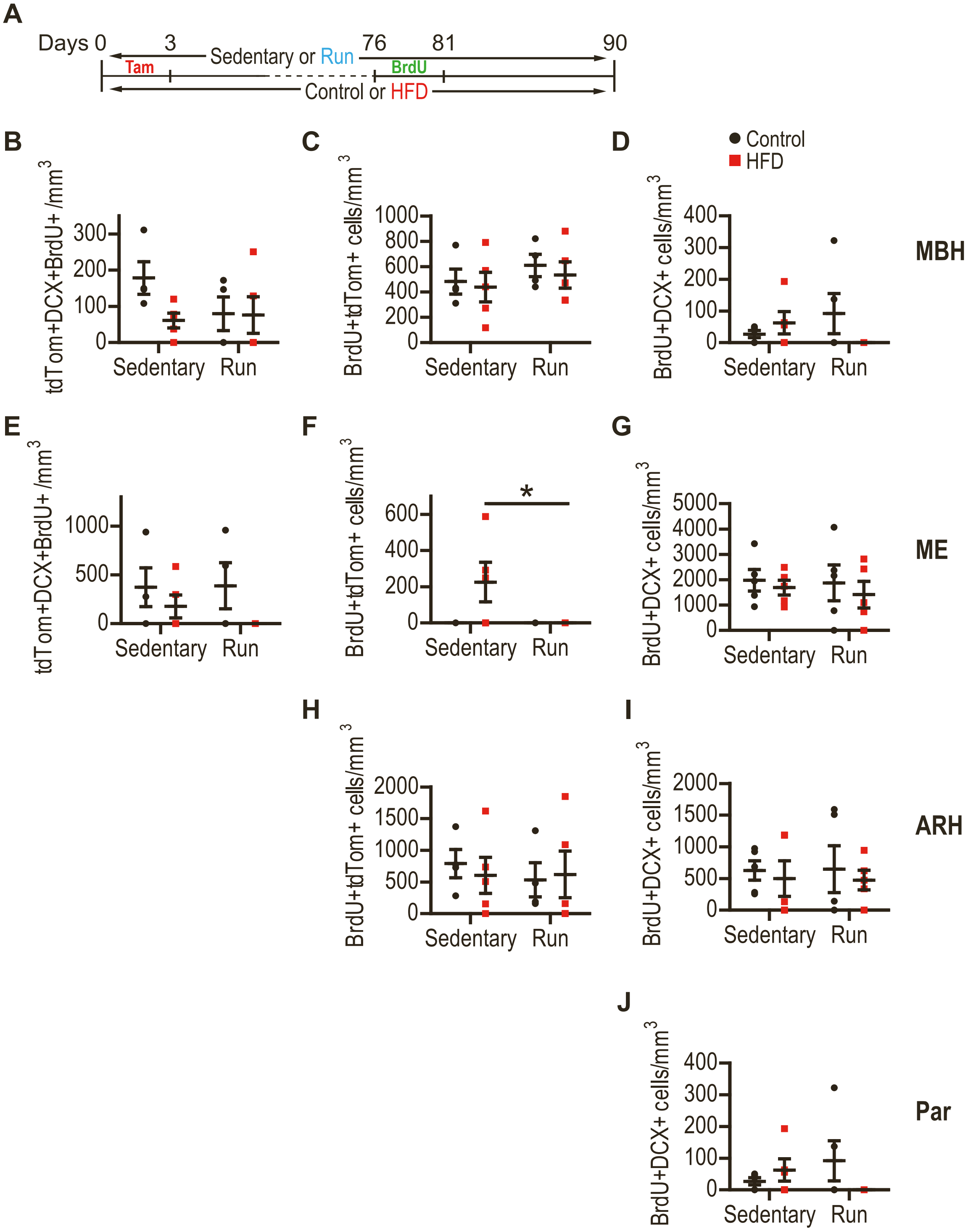


## Two-Way ANOVA results

| tdTom+DCX+BrdU+ | | | | tdTom+BrdU+ | | | | DCX+BrdU+ | | | | |
|---|---|---|---|---|---|---|---|---|---|---|---|---|
| MBH | ARH | ME | Par | MBH | ARH | ME | Par | MBH | ARH | ME | Par | |
| n.s. | *** | n.s. | *** | n.s. | n.s. | n.s. | * | n.s. | n.s. | n.s. | n.s. | Exercise |
| n.s. | * | n.s. | *** | n.s. | n.s. | n.s. | n.s. | n.s. | n.s. | n.s. | n.s. | Diet |
| n.s. | ** | n.s. | *** | n.s. | n.s. | n.s. | n.s. | n.s. | n.s. | n.s. | n.s. | Interaction |

**Figure S5. Long-term running or HFD do not alter the density of BrdU+DCX+ cells, related to Figure 5**
(A) A schematic of the protocol.
(B, E) Density of tdTom+DCX+BrdU+ cells in the MBH (B) and the ME (E).
(C, F, H) Density of BrdU+tdTom+ cells in the MBH (C), the ME (F) and the ARH (H).
(D, G, I, J) Density of BrdU+DCX+ cells in the MBH (D), the ME (G), the ARH (I) and the parenchyma (J).
The table depicts statistical significance in all 4 hypothalamic compartments. Two-Way ANOVA: *$p < 0.05$, **$p < 0.01$, ***$p < 0.001$.
Data are presented as mean ± SEM.

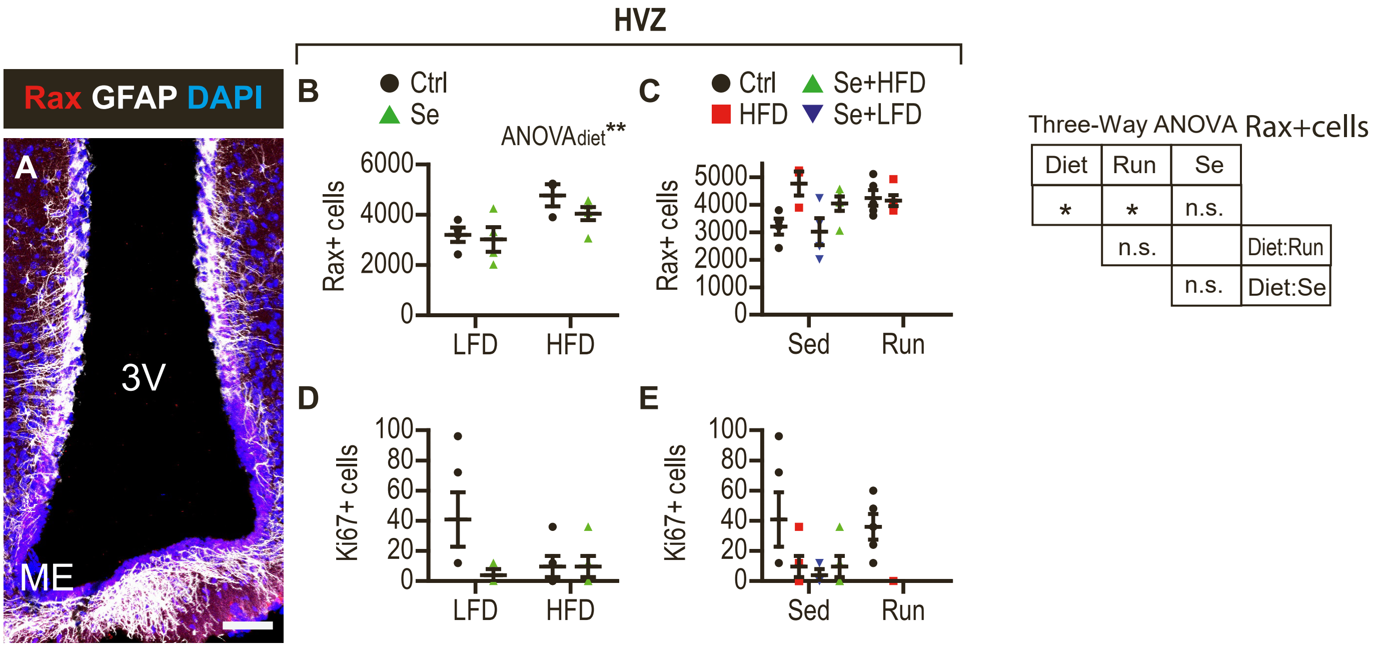


**Figure S6. Selenium does not affect the number of HVZ cells, related to Figure 6**

(A) A representative confocal image of the HVZ stained as indicated.

(B-C) Quantification of RAX+ cells in the HVZ.

(D-E) Quantification of Ki67+ cells in the HVZ.

N = 5/group. S.b. = 50 μm. Data information: Two-Way ANOVA (panel B: exercise and interaction: n.s., diet $F(1,12) = 12.16$, $p = 0.0045$), Three-Way ANOVA (panel E: selenium $F(1.22) = 7.06$, $p = 0.014$). Three-Way ANOVA table for panel C. (B, D: Bonferroni’s test, C, E: Tukey test). Data are presented as mean ± SEM.

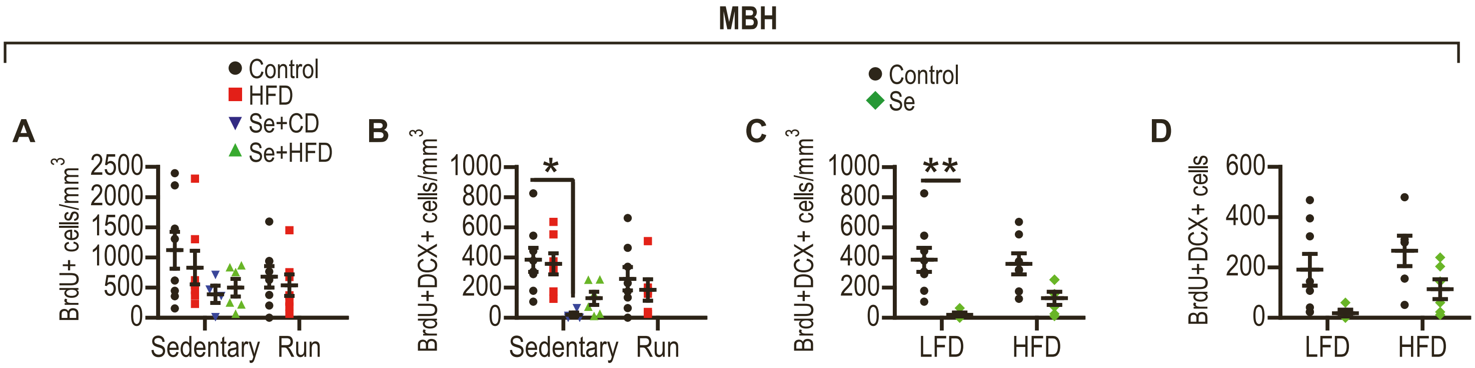


**Figure S7. Selenium reduces number of new immature neurons in the MBH, related to Figure 7**
(A-D) Quantifications of the density of BrdU+ (A) and BrdU+DCX+ cells (B-C) and the number of BrdU+DCX+ cells (D) in the MBH. N = 4-6/group. Data information: Three-Way ANOVA (panel A: selenium $F(1,34) = 4.28$, $p = 0.046$, all other: n.s.; B: selenium $F(1,33) = 14.26$, $p = 0.00063$, all other: n.s.), Two-Way ANOVA (panel C: selenium $F(1,21) = 17.26$, $p = 0.0004$; D: selenium $F(1,20) = 7.86$, $p = 0.011$). A, B: Tukey test; C, D: Bonferroni's test. *$p < 0.05$, **$p < 0.01$. Data are presented as mean ± SEM.
